# The Breast Cancer Oncogene EMSY Represses Transcription of Antimetastatic microRNA miR-31

**DOI:** 10.1016/j.molcel.2014.01.029

**Published:** 2014-03-06

**Authors:** Emmanuelle Viré, Christina Curtis, Veronica Davalos, Anna Git, Samuel Robson, Alberto Villanueva, August Vidal, Isaia Barbieri, Samuel Aparicio, Manel Esteller, Carlos Caldas, Tony Kouzarides

**Affiliations:** 1 Gurdon Institute and Department of Pathology, University of Cambridge, Tennis Court Road, Cambridge CB2 1QN, UK; 2 Department of Oncology and Cancer Research UK, Cambridge Research Institute, Li Ka Shing Centre, Robinson Way, CB2 0RE, UK; 3 Cancer Epigenetics and Biology Program (PEBC), Bellvitge Biomedical Research Institute (IDIBELL), Barcelona 08907, Spain; 4 Department of Pathological Anatomy, Bellvitge University Hospital, Bellvitge Biomedical Research Institute (IDIBELL), Barcelona 08907, Spain; 5 Department of Molecular Oncology, British Columbia Cancer Agency, 675 West 10^th^ Avenue, V5Z 1L3 Vancouver, Canada; 6 Cambridge Breast Unit, Addenbrooke’s Hospital, Cambridge University Hospital, NHS Foundation Trust and NIHR Cambridge Biomedical Research Centre, Cambridge CB2 2QQ, UK; 7 Cambridge Experimental Cancer Medicine Centre (ECMC), Cambridge CB2 0RE, UK

## Abstract

Amplification of the *EMSY* gene in sporadic breast and ovarian cancers is a poor prognostic indicator. Although EMSY has been linked to transcriptional silencing, its mechanism of action is unknown. Here, we report that EMSY acts as an oncogene, causing the transformation of cells in vitro and potentiating tumor formation and metastatic features in vivo. We identify an inverse correlation between *EMSY* amplification and miR-31 expression, an antimetastatic microRNA, in the METABRIC cohort of human breast samples. Re-expression of miR-31 profoundly reduced cell migration, invasion, and colony-formation abilities of cells overexpressing EMSY or haboring *EMSY* amplification. We show that EMSY is recruited to the miR-31 promoter by the DNA binding factor ETS-1, and it represses miR-31 transcription by delivering the H3K4me3 demethylase JARID1b/PLU-1/KDM5B. Altogether, these results suggest a pathway underlying the role of EMSY in breast cancer and uncover potential diagnostic and therapeutic targets in sporadic breast cancer.

## Highlights

• EMSY is an oncogene that represses miR-31 transcription in a BRCA2-independent manner
• Re-expression of miR-31 abrogates EMSY-mediated effects on cell migration in vitro
• EMSY and miR-31 levels anticorrelate in human breast samples from the METABRIC cohort
• EMSY, ETS-1, and KDM5B co-occupy miR-31 promoter and repress its transcription

## Graphical Abstract

**Figure:**
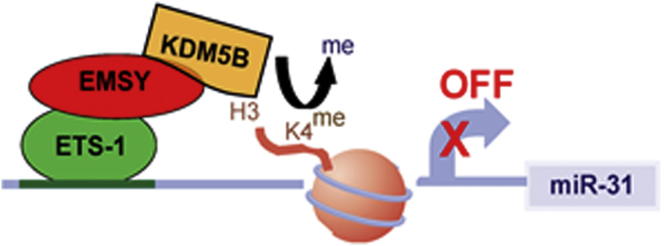

## Teaser

EMSY gene amplification is an indicator of breast and ovarian cancers. Viré et al. report that EMSY is an oncogene that functions by repressing the transcription of antimetastatic miR-31. Repression involves the transcription factor ETS-1 and the histone demethylase KDM5B. miR-31 is a key player in cancer-related EMSY function because miR-31 expression reduces EMSY-mediated oncogenicity.

## Introduction

The amplification of *EMSY* has been found in 17% and 13% of sporadic ovarian and breast cancers, respectively, and it is associated with a poor outcome (Brown et al., 2006, 2008, 2010; Hughes-Davies et al., 2003; Raouf et al., 2005; Rodriguez et al., 2004). The *EMSY* (c11orf30) gene maps to 11q13-11q14, a locus that harbors several known and potential oncogenic drivers frequently amplified in breast cancer, most notably in the estrogen-receptor-positive (ER+) luminal subtype. EMSY was shown to silence BRCA2 transcriptional activity and localize to sites of repair after DNA damage (Hughes-Davies et al., 2003). Although the precise cellular function of EMSY remains unknown, numerous lines of evidence suggest that EMSY plays a role in transcriptional regulation. First, the N terminus of EMSY has an evolutionarily conserved EMSY N-terminal domain that structurally ressembles the DNA binding domain of homeodomain proteins (Chavali et al., 2005). Second, EMSY binds chromatin-regulating factors such as HP1 (Ekblad et al., 2005; Hughes-Davies et al., 2003). Third, EMSY was found as a component of multiprotein complexes linked to transcriptional control in *D. melanogaster* (Garapaty et al., 2009). Furthermore, EMSY was involved in the repression of interferon (IFN)-stimulated genes in a BRCA2-dependent manner (Ezell et al., 2012). Finally, recent reports identify EMSY as part of the Nanog interactome (Costa et al., 2013) as well as the SIN3B complexome (Malovannaya et al., 2011).

MicroRNAs (miRNAs) are conserved small noncoding RNAs that function as key negative regulators of gene expression. Over the past years, a number of miRNAs were found to behave as tumor suppressor genes or oncogenes (Garzon et al., 2009, 2006; Iorio et al., 2005). Classically, miRNAs are defined as tumor suppressors gene when they target an oncogene. Recent studies have provided evidence for widespread deregulation of miRNA in cancer leading to cell invasion, migration, and metastasis (Bullock et al., 2012; Mirnezami et al., 2009; Visone and Croce, 2009). Like other human genes, miRNA expression can be altered by several mechanisms, such as chromosomal abnormalities, mutations, defects in their biogenesis machinery, epigenetic silencing, or the deregulation of transcription factors (TFs) (Kozaki et al., 2008; Lujambio et al., 2008; Saito et al., 2006).

Here, we identify several miRNAs whose expression varies with EMSY expression levels. Among these, we find that miR-31, an antimetastatic microRNA involved in breast cancer (Valastyan et al., 2009a), is repressed by EMSY. Chromatin immunoprecipitation (ChIP) experiments show that EMSY binds to the miR-31 promoter. We demonstrate that the TF ETS-1 recruits EMSY and that EMSY binding correlates with JARID1b/PLU-1/KDM5B occupancy at the target promoter. Altogether, our results identify EMSY as a regulator of miRNA gene expression and provide insights into the molecular mechanisms by which EMSY contributes to the initiation or progression of breast cancer.

## Results

### EMSY Exhibits All the Hallmarks of an Oncogene

**Figure 1:**
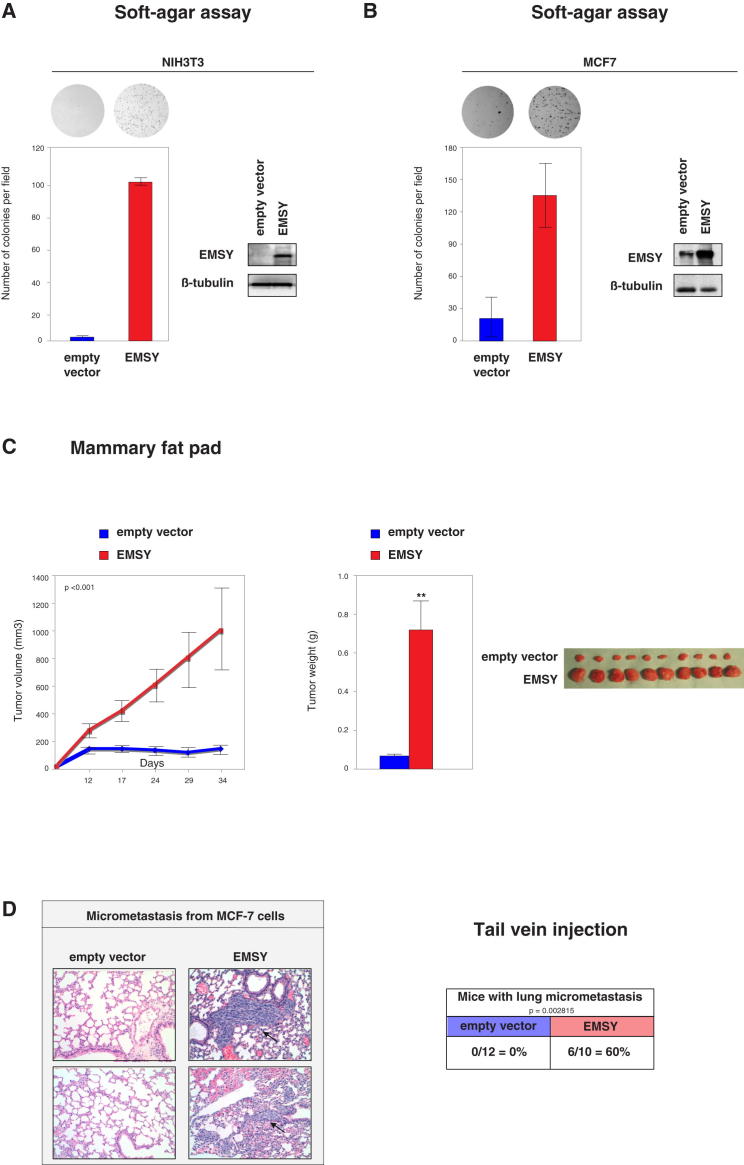
EMSY Overexpression Results in Oncogenic Transformation (A) EMSY increases anchorage-independent growth in soft-agar assays. NIH 3T3 cells stably transfected with human *EMSY* gene or an empty vector were grown in soft agar for 14 days. Error bars represent SD of the mean. Top, representative pictures of the colonies. Right, representative western blot analysis of EMSY expression. Each value is the average of three independent experiments, and error bars display the SD of the mean. (B) Soft-agar assay for MCF-7 cells stably overexpressing EMSY or the empty vector. Results are presented as in (A). Each value is the average of three independent experiments, and error bars display the SD of the mean. (C) MCF-7-EMSY cells were injected into the mammary fat pad of 5-week-old female athymic nu/nu mice (n = 10). Mice were sacrificed 34 days after injection, and then tumors were excised and weighed. Quantifications of tumor volume (left) and weight (middle) are shown. Right, representative photographs of the tumors. Error bars display the SD of the mean. (D) MCF-7-EMSY cells induce micrometastasis in the lungs after injection into the tail vein of 5-week-old female athymic nu/nu mice (n = 10). Mice were sacrificed 62 days after injection. Left, metastasis was analyzed macroscopically and by H&E tissue staining. Representative photographs of the micrometastasis are shown, and arrows indicate the micrometastasis. Right, quantifications of the micrometastasis obtained. Error bars display the SD of the mean. See also Figure S1.

To determine the basis of EMSY’s contribution to breast cancers, we asked whether EMSY can induce malignant transformation. First, we tested this by stably transfecting mouse immortalized fibroblast NIH 3T3 cells with the full-length human *EMSY* coding sequence (Figure S1A available online). Figure 1A shows that EMSY overexpression significantly confers the ability to NIH 3T3 fibroblasts to form colonies in soft agar. Then, given that *EMSY* amplifications are observed predominantly in luminal breast tumors, we engineered the luminal breast cancer MCF-7 cell line, which harbors normal levels of EMSY, to stably overexpress EMSY (MCF-7-EMSY) and similarly tested the oncogenic activity of several clones using the soft-agar assay. We confirmed EMSY overexpression by western blotting and quantitative real-time PCR (qRT-PCR; Figures S1B and 1B). Figure 1B shows that EMSY overexpression significantly enhances anchorage-independent growth of MCF-7 cells. This effect is unlikely to be due to an increase in proliferation, given that no differences in growth rate could be detected between the MCF-7 and MCF-7-EMSY cell lines (Figure S1C).

To further investigate the tumorigenic capacity of EMSY, we set out to determine whether EMSY affects tumor growth in vivo. To this end, equal numbers of mock- and EMSY-expressing MCF-7 cells were orthotopically implanted into the mammary fat pad of athymic nu/nu mice, and the animals were monitored twice a week over 34 days. MCF-7 cells are poorly invasive and metastatic, but EMSY-transformed cells produced tumors first measurable after 12 days and that continued to grow in size until the termination of the experiment. In contrast, no tumors were detected in MCF-7 control cells (Figure 1C). Finally, EMSY was demonstrated to increase the metastatic potential of MCF-7 cells, given that mice injected in the tail vein with MCF-7-EMSY cells developed more lung micrometastases than those injected with controls cells. Histological staining of the lungs were observed and micrometastasis were quantified (Figure 1D). Altogether, these results establish EMSY as a potent breast cancer oncogene in vitro and in vivo.

### EMSY Affects miRNA Expression

**Figure 2:**
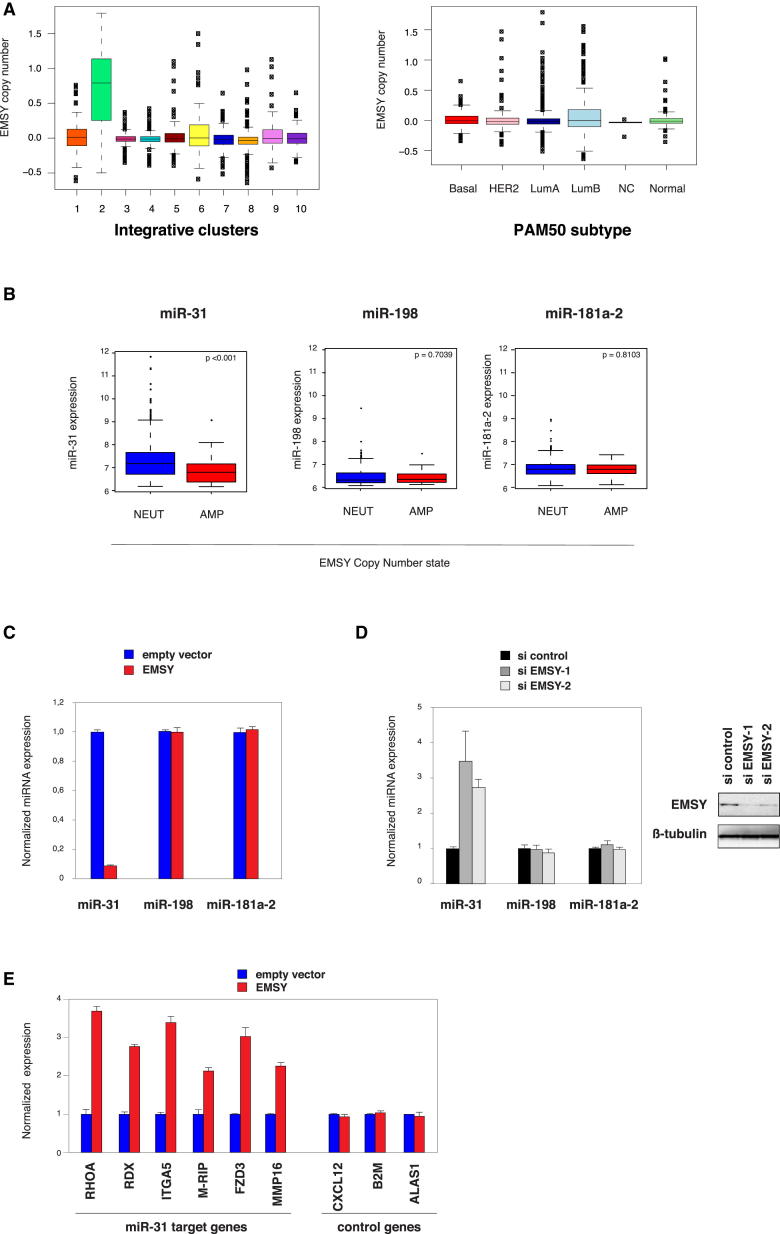
Expression of EMSY Anticorrelates with miR-31 Levels In Vitro and In Vivo (A) Box plots illustrating the distribution of EMSY copy-number-segmented means across the integrative subgroups (left) and PAM50 intrinsic subtypes (right) for 1,283 tumors from the METABRIC cohort (see also Table S1). (B) Box plot of miR-31 expression for cases with *EMSY* amplification (AMP; n = 45, red boxes) versus neutral copy numbers (NEUT; n = 809, blue boxes). The p value was determined by a Wilcoxon rank-sum test. (C) EMSY represses miR-31 transcription. qRT-PCR analysis of mature forms of the indicated miRNAs in MCF-7 cells stably overexpressing EMSY or control. Error bars represent SD of the mean. (D) Left, relative expression levels of miR-31, miR-198, and miR-181-a2 determined by qRT-PCR in MCF-7 cells transfected with siRNAs targeting human EMSY. Error bars represent SD of the mean. Right, representative western blot analysis of EMSY expression in MCF7 cells transfected with siRNAs targeting human EMSY. (E) Relative expression of the miR-31-regulated prometastatic genes upon EMSY overexpression in MCF-7 cells. Transcripts levels were measured by qRT-PCR. Error bars represent SD of the mean. See also Figure S2.

In order to understand the mechanisms by which EMSY induces oncogenic transformation, and given its potential to regulate transcription (Hughes-Davies et al., 2003), we sought to identify genes regulated by EMSY. We considered the possibility that EMSY may regulate the expression of critical miRNAs, given that certain miRNAs are associated with poor breast cancer outcome, similar to *EMSY* amplification (Garzon et al., 2009; Iorio and Croce, 2009; Iorio et al., 2005; Lujambio et al., 2008). First, we performed an unbiased screen to identify miRNAs whose expression may vary with *EMSY* levels. Using a qPCR-based array, we profiled the expression of 88 miRNAs known or predicted to alter their expression during breast cancer initiation and/or progression in MCF-7 cells depleted from EMSY using small interfering RNA (siRNA). We found 38 significantly deregulated miRNAs (9 downregulated and 29 upregulated; Table S1). Then, we asked whether these potential EMSY targets would also be deregulated in tumors where *EMSY* is amplified. Thus, we investigated miRNA expression within the Molecular Taxonomy of Breast Cancer International Consortium (METABRIC) cohort of human breast cancer samples (Curtis et al., 2012) (Table S2). The integrated analysis of genomic copy number and gene expression revealed an ER+ subgroup (IntClust 2; 3.6% of cases) with *cis*-acting aberrations at the 11q13-11q14 locus (containing *EMSY*) and extremely poor outcome. In line with our previous findings (Hughes-Davies et al., 2003), the 11q13-11q14 *cis*-acting group (IntClust 2; Figures 2A and S2A) was enriched for *EMSY* amplification. *EMSY* copy-number alterations occur predominantly in ER+ tumors (91.13% of the *EMSY* amplified cases are ER+; 7.59% of the *EMSY* amplified cases are ER−; 73.4% of the entire cohort is ER+; IntClust 2; Figure 2A). The miRNA expression levels were available for 1,283 samples from this cohort. We compared *EMSY*-amplified versus neutral cases from the METABRIC data set and found that 12 miRNAs were significantly deregulated (six downregulated and six upregulated; Benjamini-Hochberg adjusted p value < 0.05; Table S3). Given that miR-31 was the only common target identified from these two approaches (downregulated in *EMSY* amplified cases and upregulated in cells depleted from EMSY) and that miR-31 has been previously reported as a microRNA involved in the suppression of metastasis in breast cancer (Valastyan et al., 2009a, 2009b, 2010, 2011; Valastyan and Weinberg, 2010, 2011), we considered the possibility that miR-31 is an EMSY target gene and that it may be part of the mechanism underpinning EMSY’s oncogenic potential. Therefore, we decided to focus our interest on this microRNA.

Next, we set out to further confirm the observation that miR-31 expression levels are significantly lower in *EMSY*-amplified versus neutral tumors (Figure 2B; p value < 0.001; Wilcoxon rank-sum test). Given that the expression probe for *EMSY* on the Illumina HT12 v3 array was nonresponsive, we profiled miR-31 expression levels by qRT-PCR in a representative subset of 98 primary tumors from the METABRIC cohort. This analysis corroborates our previous findings, given that *EMSY* copy-number and expression levels were highly correlated (ρ = 0.795, Spearmann’s rho; p value < 0.0001). We found that *EMSY* expression strongly anticorrelated with miR-31 expression (ρ = −0.732, Spearmann’s rho; p value < 0.0001; Figures S2B and S2C). Then, we further examined the relationship between EMSY and miR-31 expression levels. The expression of miR-31 was monitored by qRT-PCR in MCF-7 cells overexpressing EMSY and in comparison to control cells. This analysis revealed that miR-31 levels were significantly reduced in cells stably overexpressing EMSY, whereas the expression of other miRNAs such as miR-181a-2 and miR-198 remained unchanged (Figure 2C). Conversly, and consistent with these findings, miR-31 was markedly upregulated in EMSY-depleted MCF-7 cells (Figure S2E), in comparison to miR-181a-2 and miR-198 (Figure 2D). Finally, we evaluated miR-31 levels in tumors obtained from the mammary fat pad experiment presented in Figure 1C. Figure S2D shows that miR-31 expression is lower in tumors generated from the MCF-7-EMSY than from MCF-7 control cells. Altogether, these results support an inverse correlation between EMSY and miR-31 expression levels.

Given that miR-31 affects cell invasion and migration via its pleiotropic regulation of prometastatic target genes (Valastyan et al., 2009b), we tested whether EMSY expression impacts on miR-31 target genes. To test this, we analyzed MCF-7 cells stably overexpressing EMSY (Figures 1B and S1B) for the expression of a set of validated miR-31 target genes (Valastyan et al., 2009a, 2009b). Figure 2E shows that MCF-7-EMSY cells display higher levels of transcripts reported to be under the control of miR-31 (RhoA, RDX, ITGA5, M-RIP, FZD3, and MMP16) in comparison to control cells. This effect is specific, given that non-miR-31 target transcripts, namely B2M, CXCL12, and ALAS1, remain unaffected (Figure 2E). Three miR-31 target genes, namely ITGA5, RDX, and RhoA, are crucial for the antimetastatic response of miR-31 (Valastyan et al. 2009a, 2009b).

### miR-31 Reverts EMSY Oncogenicity In Vitro

**Figure 3:**
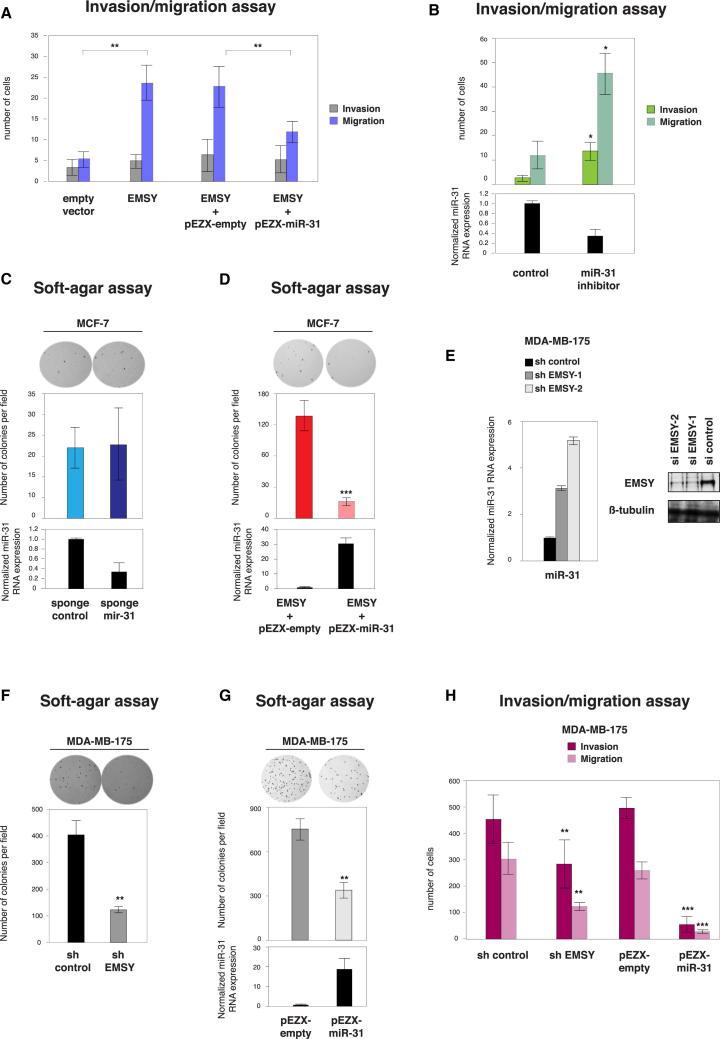
miR-31 Reverts EMSY Oncogenicity In Vitro (A) Invasion and migration assay of MCF-7 cells stably expressing EMSY. Reintroduction of miR-31 reduces EMSY-mediated effects. Error bars represent SD of the mean. (B) Invasion and migration assay of MCF-7 cells transiently depleted from miR-31 using oligonucleotides. Bottom, relative expression levels of miR-31 measured by qRT-PCR. (C) Stable depletion of miR-31 reduces anchorage-independent growth of MCF-7 cells. Error bars represent SD of the mean. (D) Soft-agar assay of MCF-7 cells stably overexpressing EMSY after the reintroduction of miR-31. Results are presented as in (C). (E) The expression levels of the indicated miRNAs were measured by qRT-PCR in MDA-MB-175, harouring *EMSY* amplification, and stably depleted from EMSY. Error bars represent SD of the mean. (F) Soft-agar assay of MDA-MB-175 cells stably depleted from EMSY. (G) Exogenous expression of miR-31 reduces anchorage-independent growth of of MDA-MB-175. Results are presented as in (C). (H) Invasion and migration assay of MDA-MB-175 cells either stably depleted from EMSY or stably overexpressing miR-31. Error bars represent SD of the mean. ^∗^p < 0.05, ^∗∗^p < 0.01, and ^∗∗∗^p < 0.001. See also Figure S3.

Then, we set out to test whether EMSY overexpression impacts on the invasive and migrative capability of MCF-7 cells. Using a Boyden chamber assay, we observed that the overexpression of EMSY increases MCF-7 migrative capacity when compared to control cells (Figure 3A). These findings are consistent with the results obtained from the mammary fat pad and tail vein injection experiments described in Figure 1. Then, to establish whether miR-31 mediates, at least in part, the effects of EMSY overexpression, we performed a “rescue” experiment wherein miR-31 was exogenously expressed in the context of EMSY overexpression. MCF-7 cells stably overexpressing EMSY were transfected with a vector encoding miR-31. Enforced expression of miR-31 significantly reversed EMSY-mediated induction of cell migration (Figure 3A). Given that miR-31 has been described to affect invasion and migration (Valastyan et al., 2009b, 2010), we tested whether miR-31 inhibition alters the initial acquisition of a transformed phenotype. We used antisense oligonucleotides to deplete MCF-7 cells from miR-31. We found that a loss of miR-31 significantly enhanced the invasion and migration capacity of MCF-7 cells (Figure 3B). However, when miR-31 sponges were used to stably deplete miR-31 from MCF-7 cells, we observed that the resulting cells formed colonies in soft agar as efficiently as control MCF-7 cells (Figure 3C). Thus, the loss of miR-31 from MCF-7 cells did not enhance transformation. These data indicate that EMSY overexpression induces MCF-7 cell transformation, and, in a second step, EMSY functions to downregulate miR-31, leading to the progression of the transformed phenotype (i.e., the acquisition of traits such as invasion and migration). Importantly, these data are completely consistent with those reported by Valastyan et al. (2009b) and led us to propose a model for the EMSY-miR-31 interaction. Then, we asked whether the re-expression of miR-31 in MCF-7 cells stably overexpressing EMSY could abrogate EMSY-induced oncogenic activity using the soft-agar assay. Figure 3D shows that miR-31 re-expression profoundly reduces the ability of the MCF-7-EMSY cells to form colonies in soft agar.

Next, having shown that EMSY and miR-31 levels inversely correlate in human breast cancers, we investigated whether the EMSY-miR-31 interaction is also seen in cell lines harboring amplification of the *EMSY*-11q13-11q14 locus. We used two such cell lines, MDA-MB-175 and MDA-MB-415 (Rodriguez et al., 2004), and confirmed that both have high EMSY and very low miR-31 levels in comparison to MCF-7 cells (Figure S3A). Figure 3E shows that miR-31 was markedly upregulated in MDA-MB-175 cells upon EMSY depletion, consistent with our results in MCF-7 cells (Figure 2C). Similar results were obtained in MDA-MB-415 cells (Figure S3B). Then, we tested the oncogenic activity of these cells in vitro using the soft-agar assay. EMSY depletion significantly reduces the anchorage-independent growth of both cell lines (Figures 3F and S3D). Moreover, increased expression of miR-31 phenocopies the effect of EMSY knockdown in these two EMSY-amplified cell lines (Figures 3G and S3E). Finally, we monitored the invasion and migration abilities of MDA-MB-175 and MDB-MB-415 cells after the depletion of EMSY or overexpression of miR-31. EMSY depletion significantly reduced the invasion and migration abilities of the cell lines (Figures 3H and S3F). Exogenous expression of miR-31 reduced the invasive and migrative rates of both cells lines (Figures 3H and S3F). Therefore, we conclude that miR-31 expression phenocopies the effect of EMSY depletion. Altogether, these results indicate that the invasion and migration features of *EMSY*-amplified breast cancer cells are dependent on EMSY levels. These results also demonstrate that miR-31 is an important antagonist of EMSY’s function in breast cancer.

### EMSY Represses miR-31 Transcription and Binds to the miR-31 Promoter

**Figure 4:**
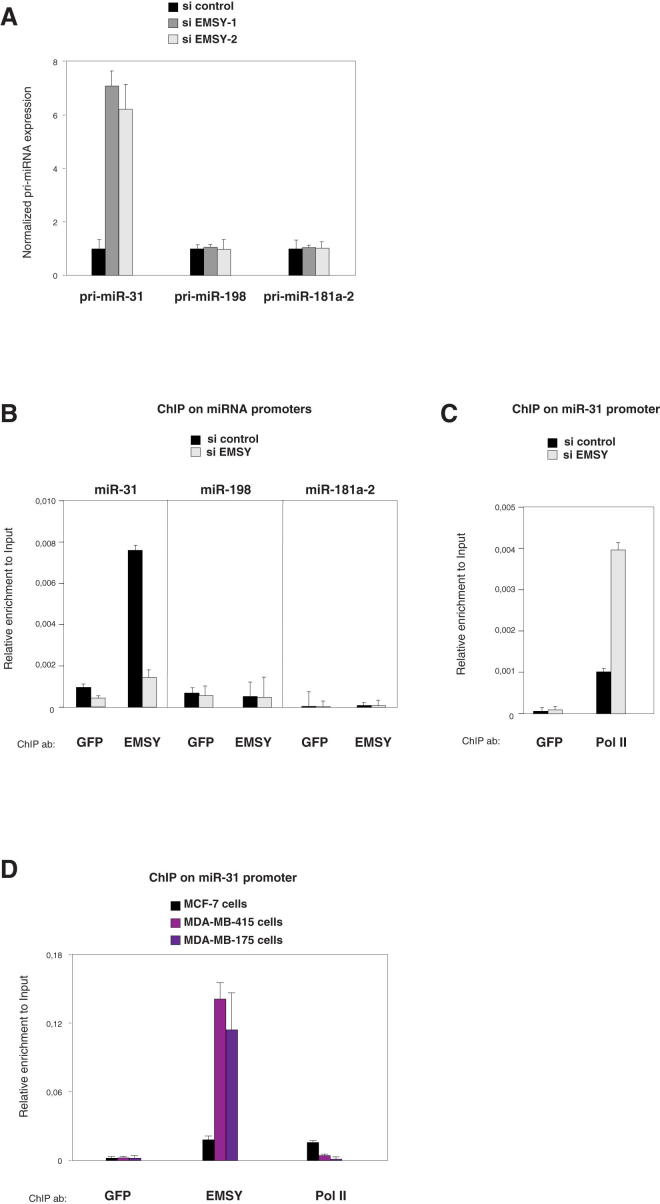
EMSY Represses miR-31 Transcription and Associates to miR-31 Promoter (A) pri-miRNAs level determined by qRT-PCR in MCF-7 cells transiently depleted from EMSY. Error bars display SD of the mean. (B) EMSY associates to miR-31 promoter. ChIP in MCF-7 cells treated with control siRNA or EMSY siRNA using anti-EMSY or anti-GFP (control). The data have been normalized to “input DNA” amplification levels for each amplicon. Each amplicon was analyzed in duplicate. Experiments were repeated at least two times with similar results. A representative example of a ChIP analysis is shown. Error bars display the SD of the mean. (C) EMSY depletion leads to increased levels of RNA polymerase II at miR-31 promoter. ChIP with anti-RNA polymerase II in MCF-7 cells treated with control siRNA or EMSY siRNA. Results are presented as in (B). (D) EMSY and RNA polymerase II occupancy at the miR-31 promoter were assessed in MCF-7, MDA-MB-175, and MDA-MB-415 cells. Results are presented as in (B). See also Figure S4.

EMSY is not listed as a putative miR-31 target, and its expression remained constant upon miR-31 transfection (data not shown). In contrast, the depletion of EMSY led to increased levels of primary miR-31 transcripts (pri-miR-31; Figure 4A) suggesting that EMSY affects the transcription of the *miR-31* gene rather than the processing of its transcripts. These observations prompted us to investigate whether EMSY directly represses transcription of miR-31 by binding to its promoter region. We identified the promoter of miR-31 using 5′ rapid amplification of complementary DNA (cDNA) ends (RACE) experiments (Figure S4A). ChIP analyses indicated that EMSY associated with the promoter of miR-31 but did not bind the regions upstream of two control miRNA genes, miR-181a-2 and miR-198 (Figures 4B and S4B). Furthermore, we found that RNA polymerase II occupancy on the miR-31 promoter increased upon the downregulation of EMSY, consistent with EMSY repressing transcription (Figure 4C). Finally, to address whether EMSY amplification could further repress miR-31, as predicted by our previous results, we compared EMSY and RNA polymerase II occupancy between MCF-7 cells (which exhibit normal EMSY levels) and MDA-MB-415 and MDA-MB-175 cells (which exhibit amplification of the EMSY gene). EMSY occupancy on the miR-31 promoter was higher in cells with EMSY amplification, whereas RNA polymerase II occupancy was lower (Figure 4D). Altogether, these observations indicate that EMSY associates with the promoter of miR-31 and represses its expression.

### The Transcription Factor ETS-1 Recruits EMSY to the miR-31 Promoter

**Figure 5:**
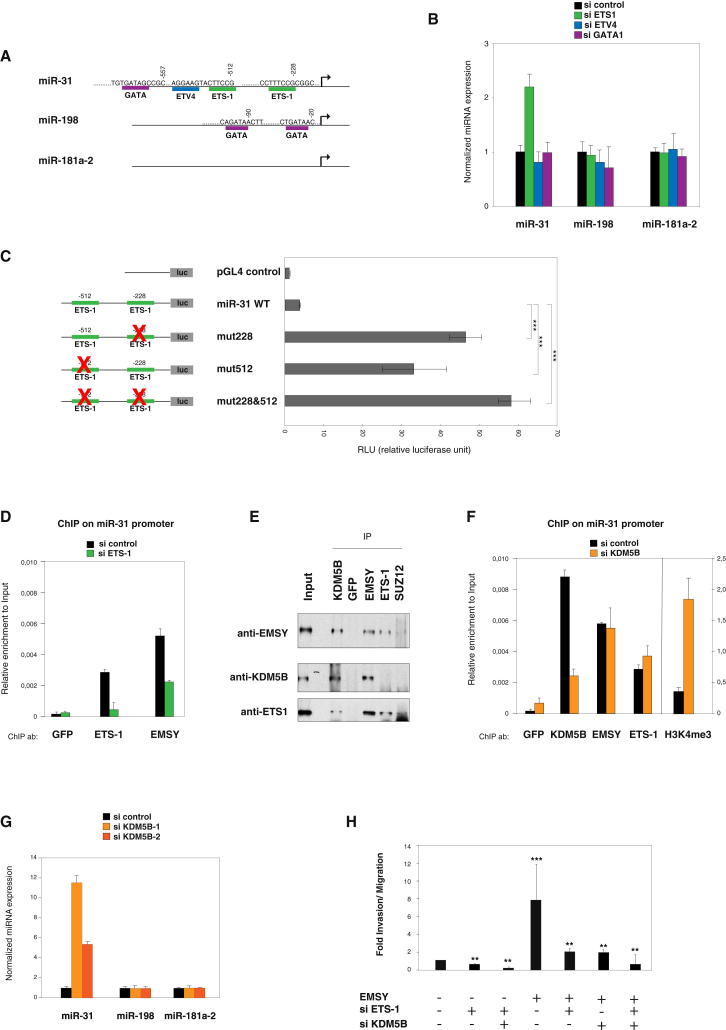
EMSY Is Recruited to the miR-31 Promoter via ETS-1 Transcription Factor and Interacts with H3K4me3 Histone Demethylase KDM5B (A) Schematic view of the indicated miRNA promoter region (1,000 bp upstream from the putative transcription start site; arrow). The locations of predicted putative TF binding sites are shown for ETS-1, ETV4 (PEA3), and GATA1. Numbering is relative to the transcription start site. (B) Expression of the *miR-31*, *miR-198*, and *miR-181a-2* genes were studied by qRT-PCR after cells were treated with control, ETS-1, ETV4 (PEA3), or GATA1 siRNAs. Error bars represent SD of the mean. (C) MCF-7 cells were transiently transfected with the *cis*-regulatory element of miR-31 promoter fused to the luciferase reporter vector pGL4, as indicated in the scheme (left). The luciferase activity of empty pGL4 was used as the control. Luciferase activities, expressed as relative luciferase units, were normalized to the level of Renilla luciferase activity and then to control RLU (empty vector). Site-directed mutagenesis was used to change the sequence of each or both of the ETS-1 binding sites. Error bars represent SD of the mean. (D) Chromatin from MCF-7 cells depleted from ETS-1 was immunoprecipitated with the indicated antibodies. Results are presented as in Figure 4B. (E) Endogenous coimmunoprecipitation assays for detecting the interaction between EMSY, ETS-1, and KDM5B in MCF-7 cells. Whole-cell extracts were immunoprecipitated with anti-ETS-1, anti-KDM5B, anti-SUZ12, and anti-EMSY or preimmune GFP used as control IgG. The presence of EMSY, ETS-1, and KDM5B in immunoprecipitates was detected by western blotting. (F) KDM5B depletion affects H3K4me3 levels on miR-31 promoter. ChIP in control and KDM5B-siRNA-treated MCF-7 cells. (G) The expression levels of mature forms of the indicated miRNAs were measured by qRT-PCR analysis in control and KDM5B-siRNA-treated MCF-7 cells. (H) Migration assay of MCF-7 cells overexpressing EMSY or an empty vector after cells were treated with siRNA against ETS-1, KDM5B, or a combination of both. Error bars represent SD of the mean. ^∗^p < 0.05, ^∗∗^p < 0.01, and ^∗∗∗^p < 0.001. See also Figure S5.

To further understand the mechanisms by which EMSY silences miR-31, we sought to decipher how EMSY is recruited to the miR-31 promoter. Given that EMSY has no obvious DNA binding domain, we considered the possibility that EMSY is recruited to the promoter of miR-31 via a DNA binding TF. To probe this hypothesis, we tested whether BRCA2, a known binding partner of EMSY (Hughes-Davies et al., 2003), may be involved in EMSY’s recruitment. The depletion of BRCA2 has no effect on miR-31 expression, indicating that BRCA2 is not directly involved in the EMSY/miR-31 pathway (Figure S4C). Then, we examined the DNA sequence upstream of the miR-31 transcription start site for TF binding sites. Figure 5A shows that putative binding sites for ETS family members (ETS-1 and ETV4/PEA3) can be found in the miR-31 regulatory region but not within the analogous regions of miR-198 and miR-181a-2, whereas a GATA1 site is present within miR-31 and miR-198. The position weight matrix (PWM) for the ETS-1 TF binding motif was identified from the JASPAR database (http://jaspar.genereg.net). The presence of the ETS-1 binding motif in the miRNA promoters was confirmed with the matchPWM function in the Biostrings package with a minimum matching score of 100%. Importantly, ETS-1 is a classic DNA binding protein implicated in transcriptional regulation, and its expression correlates with higher incidence of metastasis and poorer prognosis in breast and ovarian carcinoma (Buggy et al., 2004; Furlan et al., 2008; Gilles et al., 1997). In order to validate the TF binding site predicions, we depleted MCF-7 cells for each of the TFs with a putative binding site in the miR-31 promoter (ETS-1, ETV4, and GATA1) and assessed the expression of miR-31. Figures 5B and S5A show that, of the three TFs, only ETS-1 downregulation led to the specific upregulation of miR-31. Importantly, it did so without affecting the expression of miR-198 and miR-181a-2. To further confirm these results and validate the predictions from the bioinformatic analyses, we tested the ETS-1 motifs in the miR-31 *cis*-regulatory element in functional transcription assays. We constructed reporter vectors containing the miR-31 promoter sequence upstream of the firefly luciferase cDNA using the pGL4 plasmid. Figure 5C shows promoter activity for the miR-31 promoter region containing the 2 ETS-1 motifs. Site-directed mutagenesis of each of the ETS-1 sites (site 218, TTTCCG was mutated into TTTTTT, and site 512, CTTCCG was mutated into TTTTTT) led to a significant increase of promoter activation (Figure 5C). These results demonstrate that each of the ETS-1 binding sites are relevant to miR-31 expression and that ETS-1 functions as a repressor in this context. The mutation of both ETS-1 binding sites (218 and 512) did affect miR-31 promoter activity more than each of the single mutants. Importantly, we confirmed by ChIP that the ETS-1 protein binds directly to the miR-31 promoter within the region containing the predicted ETS binding site (Figure 5D). This analysis also showed that ETS-1 did not bind to other regions within the miR-31 promoter, nor did it bind to the promoters of miR-181a-2 or miR-198 (Figure S5C). Furthermore, depletion of ETS-1 resulted in reduced binding of both ETS-1 and EMSY to the miR-31 promoter (Figure 5D) without affecting the levels of EMSY protein in MCF-7 cells (Figure S5B). Notably, downregulation of EMSY does not affect the binding of ETS-1 to the miR-31 promoter, supporting a model in which ETS-1 recruits EMSY to the miR-31 promoter.

### The Histone H3K4me3 Demethylase KDM5b Interacts with EMSY and Contributes to miR-31 Silencing

EMSY has been found to be a component of complexes involved in transcriptional control that contain, among other proteins, members of the histone H3K4me3 demethylase family (Barrett et al., 2002, 2007; Moshkin et al., 2009). Given that JARID1b/PLU1/KDM5B has been shown to be expressed in 90% of invasive ductal carcinomas and that KDM5B represses transcription by demethylating H3K4 (Barrett et al., 2002, 2007; Lu et al., 1999; Yamane et al., 2007), we examined whether KDM5B associates with EMSY. Figure 5E shows that KDM5B coimmunoprecipitates with endogenous EMSY from MCF-7 cell extracts. Moreover, ChIP experiments showed that KDM5B binds to the miR-31 promoter at the same position as EMSY and ETS-1 (Figures 5F and S5E). Consistent with its role as a histone demethylase, the depletion of KDM5B (Figure S5D) resulted in an increase of H3K4 trimethylation on the miR-31 promoter (Figure 5F) and a concomitant increase in the expression of miR-31 (Figure 5G). Depletion of KDM5B did not affect the association of either EMSY or ETS-1 to the miR-31 promoter but did affect its own binding in that region (Figure 5F).

Finally, we asked whether ETS-1 and KDM5B contribute to EMSY’s capacity to increase the migration of MCF-7 cells. Using the Boyden chamber assay, we observed that the increased migrative ability of MCF-7 cells overexpressing EMSY is significantly reduced when cells are depleted for ETS-1, KDM5B, or a combination of both ETS-1 and KDM5B (Figure 5H). Altogether, these results support a model whereby ETS-1 directly binds to an ETS binding motif within the miR-31 promoter to recruit EMSY and KDM5B to repress transcription.

## Discussion

The *EMSY* gene is amplified and overexpressed in a substantial proportion of sporadic cases of poor prognosis breast cancer. However, determining the direct contribution of *EMSY* to breast cancer has been difficult because the gene resides within the 11q13-11q14 locus, containing a cassette of genes, including several known and potential breast cancer drivers (*CCND1*, PAK1, and *RSF1*). This has meant that *EMSY*’s oncogenic contribution has been difficult to assess. Our previous studies have shown that the EMSY protein interacts with BRCA2 and has a role in chromatin remodeling (Hughes-Davies et al., 2003). The data presented here show that EMSY itself possesses the hallmarks of an oncogene: ectopic expression of EMSY transforms NIH 3T3 fibroblasts, confers anchorage-independent growth to MCF-7 cells in soft agar, and potentiates tumor formation and metastatic features in vivo (Figure 1). In light of the recent demonstration that amplification of the 11q13-11q14 locus is associated with a particularly high-risk outcome (Curtis et al., 2012), the results presented here support the conclusion that *EMSY* is not simply a passenger of the locus but rather a significant oncogenic driver.

Our results also identify a subset of miRNAs whose expression is affected by EMSY levels in primary breast tumor samples. One of them, miR-31, is a key regulator of breast cancer metastasis. Examination of the large METABRIC cohort highlights a negative correlation between EMSY and miR-31 expression. In breast cancer cell lines, the overexpression of EMSY reduces the expression of the *miR-31* gene, increases the expression of miR-31 target genes, and induces invasion and migration. We also find that loss of miR-31 did not enhance transformation in MCF-7 cells, suggesting a two-step process, wherein EMSY overexpression induces cell transformation and then EMSY functions to downregulate miR-31, leading to the progression of the transformed phenotype (i.e., the acquisition of traits such as invasion and migration). Moreover, restoration of miR-31 significantly inhibited the invasion, migration, and colony-formation abilities of cells overexpressing EMSY or harboring *EMSY* amplification, a result which phenocopied the effects of EMSY depletion in these cells. Thus, the regulation of the miR-31 pathway by EMSY can explain, at least in part, its oncogenic behavior and association with poor prognosis (Figure 3). Moreover, miR-31 is a critical target of EMSY in breast cancer. Our results also identify an EMSY pathway in which the BRCA2 protein does not seem to contribute. Therefore, the work presented here provides evidence for the existence of at least two distinct EMSY activities: one where EMSY acts in concert with BRCA2, and one where EMSY functions in a BRCA2-independent manner.

Our data also provide mechanistic insights into the function of EMSY at the molecular level. EMSY is recruited to the miR-31 promoter by the ETS-1 TF, and, together with the KDM5B histone demethylase, these factors repress miR-31 expression (Figure 5). The ability of EMSY to function via the recruitment of the chromatin regulator KDM5B is consistent with its previously suspected involvement in chromatin regulation (Ekblad et al., 2005; Garapaty et al., 2009; Hughes-Davies et al., 2003; Moshkin et al., 2009; Yao and Polyak, 2004). Furthermore, it is interesting to note that two components of this pathway, namely ETS-1 and KDM5B, have themselves been implicated in breast cancer (Buggy et al., 2004; Yamane et al., 2007). Of particular relevance is the fact that ETS-1 is defined as a candidate breast cancer oncogene involved in the regulation of the expression of genes involved in tumor progression and metastasis (Switzer et al., 2012) and that the expression of ETS-1 correlates with higher incidence of metastasis and poorer prognosis in breast and ovarian carcinoma (Buggy et al., 2004; Furlan et al., 2008; Gilles et al., 1997). The second factor implicated in the pathway, which we identified as KDM5B, is an H3K4me2- and H3K4me3-specific demethylase belonging to the Jmjc-domain-containing family of histone demethylase. KDM5B has been shown to be overexpressed in several cancers, such as breast, prostate, and lung cancer, and is required for mammary tumor formation in xenograft mouse models (Catchpole et al., 2011; Yamane et al., 2007).

The pathway indentified here (EMSY/ETS1/KDM5B/miR-31) may not provide the unique and complete molecular explanation for the association between *EMSY* amplification and poor prognosis. EMSY’s contribution to tumorigenesis may not be solely due to the silencing of miR-31, given that other EMSY unidentified target genes may also play a role. As such, the identification of additional EMSY mRNAs and miRNA targets will be important for understanding the complex mechanisms underlying EMSY’s role in breast cancer. Moreover, the EMSY/ETS-1/KDM5B pathway may not be the only route for miR-31 transcriptional regulation. Indeed, recent reports have identified other genetic and epigenetic axes influencing miR-31 expression (Augoff et al., 2012; Yamagishi et al., 2012). For example, in adult T cell leukemia, Polycomb proteins have been shown to contribute to miR-31 downregulation (Yamagishi et al., 2012). Interestingly, a functional interplay between KDM5B and Polycomb proteins has recently been implicated in mouse development (Albert et al., 2013). In addition, another epigenetic modification, DNA methylation, is also involved in decreasing miR-31 expression in breast cancer (Vrba et al., 2013). The interplay between EMSY and these factors, in addition to other targets and/or signals such as estrogen, and how they may contribute to malignant progression are of significant interest for future work.

The data presented here, which are derived from multiple different molecular and cellular assays as well as animal studies together with correlative studies involving human breast cancer patients, collectively and consistently support the conclusion that EMSY represses transcription of the noncoding *miR-31* gene. This pathway represents one mechanism by which EMSY exerts its oncogenic and metastatic potential in breast cancer, resulting in a poor outcome prognosis of *EMSY*-amplified breast tumors. Given the convergence of four breast-cancer-associated genes (EMSY, ETS-1, KDM5B, and miR-31) with overlapping biological roles, this pathway offers a number of avenues for therapeutic intervention.

## Experimental Procedures

The details of all siRNAs, plasmids, synthetic RNAs, primers, and antibodies used in this study are provided in in the Supplemental Information and/or are available upon request.

### Cell Culture

Mouse NIH 3T3 and human breast cancer cell lines MCF-7, MDA-MB-415, and MDA-MB-175 were purchased from ATCC and cultured in Dulbecco’s modified Eagle’s medium (DMEM; Invitrogen) supplemented with 10% fetal bovine serum (FBS; Gibco) and 1% penicillin and streptomycin and grown at 37°C under 5% CO_2_.

### Xenograft Assay and Tail Vein Injection

In this study, 5-week-old female athymic nu/nu mice (Harlan Laboratories) housed under specific pathogen-free conditions were used. Mice were anesthetized and ovariectomized 2 days before the implantation of 3.0 mm pellets containing 17β-estradiol (1.7 mg/60-day release; Innovative Research of America). Cells were injected 2 days after pellet implantation. For tumor growth analysis, 4–5 × 10^6^ cells were injected in to the mammary fat pads of mice. Tumor development was monitored twice a week, and tumor width (W) and length (L) were measured. Tumor volume was estimated according to the formula V = π/6 × L × W2. Mice were sacrificed 15–30 days after injection, and then tumors were excised and weighed. For the experimental metastasis assay, 1–2 × 10^6^ cells were injected into the tail vein. Metastasis were examined later and analyzed macroscopically and by hematoxylin and eosin (H&E) tissue staining. At least ten animals were tested for each experimental condition. All experiments with mice were approved by the Bellvitge Biomedical Research Institute Animal Care and Use Committee.

### Reverse Transcription and Quantitative Real-Time PCR

Total RNA and enriched miRNA fractions were obtained with miRNeasy Mini (QIAGEN) and mirVANA kits (Ambion) according to the manufacturer’s instructions. DNase treatment was carried out in order to remove any contaminating DNA. RNA was reverse transcribed with a miScript Reverse Transcription Kit (QIAGEN) according to the manufacturer’s instructions. The real-time qRT-PCR for the quantification of miRNAs (miRNA-31, miRNA-181a-2, and miRNA-198) as well as pri-miRNAs (miR-31 pri-miRNA, miR-198 pri-miRNA, and miR-181a-2 pri-miRNA) was carried out with a miScript Primer Assays and miScript SYBR Green PCR Kit (QIAGEN) on an ABI 7500 Fast Real-Time PCR System. Transcripts of RNU5A and RNU1A small RNAs were also amplified in order to normalize the levels of miRNAs. All reverse transcriptions and no-template controls were run at the same time. qRT-PCR was used to determine the expression levels of EMSY, BRCA2, ETV4 (PEA3), GATA1, ETS-1, KDM5B, KDM5A, FZD3, MMP-16, RHOA, RDX, ITGA5, M-RIP, CXCL12, B2M, and ALAS1. B2M was amplified as an internal control in order to monitor the amount and integrity of the cDNA. All reactions were performed in duplicates. The ΔΔCt method was used for analysis. Fold change expression in a sample = 2^−ΔΔCt^. Primers sequences are available upon request. Gene expression changes are presented as relative fold change compared to the values in empty vector cells (set at 1.0).

### Real-Time-PCR-Based miRNA Expression Profiling

MCF-7 cells were analyzed for the presence and differential expression of a panel of 88 cancer-related miRNAs with cancer RT^2^ miRNA PCR arrays (RT^2^ Profiler; SABiosciences) according to the manufacturer’s instructions. Data analysis was performed with the web-based software package for the miRNA PCR array system.

### Copy Number, Gene Expression, miRNA, and Survival Analysis of Primary Tumors

In order to examine the relationship between *EMSY* amplification, miR-31 expression levels, and the expression of miR-31 targets, we employed the METABRIC cohort of ∼2,000 primary breast tumors for which paired Affymetrix SNP 6.0 copy-number data and Illumina HT-12 expression data were available. Data were processed and summarized as described by Curtis et al. (2012). The genotype and expression data (Curtis et al., 2012) are available at the European Genome-phenome Archive (http://ebi.ac.uk/ega/), which is hosted by the European Bioinformatics Institute, under accession numbers EGAS00000000083. The raw noncoding RNA microarray data (Dvinge et al., 2013) are available under accession number EGAS00000000122. Here, we report on a subset of 1,283 cases for which we also had miRNA expression profiles based on a custom Agilent platform (Dvinge et al., 2013). In brief, miRNA data were processed by iteratively removing the two most extreme outliers (on the basis of six replicates per probe) followed by cubic spline normalization. Using these data, we evaluated the relationship between *EMSY* amplification and miR-31 expression levels as well as the association between these events and clinical outcome. EMSY copy-number states were determined as previously described (Curtis et al., 2012). miR-31 expression levels were binned into low and high states on the basis of the lower and upper 15% of expression values, respectively. Statistical significance was evaluated with the log rank test. All analyses were performed in the R statistical computing language. The Wilcoxon rank-sum test was used to evaluate whether expression levels varied significantly depending on EMSY copy-number state.

### Real-time PCR of Tumor RNA

A representative subset of 98 primary tumors were selected from the METABRIC cohort in order to include cases with *EMSY* amplification (n = 28), gain (n = 20), neutral copy number (n = 30), or heterozygous deletion (n = 20). Cases were also selected to be copy-number neutral for IFNE1 and MTAP, which flank the miR-31 locus. Then, qRT-PCR was performed in order to assay EMSY and miR-31 expression levels as well as the expression of the control miRNAs, miR-191, and miR-93. Triplicate aliquots of cDNA were subjected to real-time qPCR on the ABI PRISM 7900HT system (Applied Biosystems). Relative expression values accounting for differences in amplification efficiency were calculated by automated software (SDS 2.3, Applied Biosystems) using the linear regression of a standard titration curve included for each plate. Expression was normalized for each sample by dividing the relative expression of each gene by the geometric mean of the relative expression values of multiple internal reference genes.

### 5′ RACE

RACE was carried out with an GeneRacer RLM-Race Kit (Invitrogen) according to the manufacturer’s instructions. In brief, cDNAs were made from total RNA (5 μg) with random primers according to the standard protocols. The 5′ ends of the miRNAs were amplified with a gene-specific primer. Amplicons were reamplified successively with nested-gene-specific primers. All PCRs were performed with Platinum TaqDNA Polymerase High Fidelity (Invitrogen). Amplified PCR products were purified and cloned into pCR4-Topo vectors according to TOPO-TA cloning protocols, sequenced, and analyzed by BLAST.

### Luciferase Assay

Human miR-31 promoter was amplified by PCR from genomic DNA of MCF-7 cells. PCR products were digested and ligated into pGL4.10 (luc2; Promega). Mutations were introduced by site-directed mutagenesis of the ETS-1 binding sites according to the manufacturer’s instructions for site-directed mutagenesis (Invitrogen). The pTK-luc (Renilla) vector was cotransfected with the pGL4 (firefly) vectors in order to normalize the transfection efficiency. After 48 hr of transfection, the promoter activity was measured with a Dual-Glo Luciferase Assay System (Promega) according to the manufacturer’s protocol.

### ChIP Analysis

Chromatin was prepared from MCF-7, and immunoprecipitations were performed as described previously (Dawson et al., 2009) and with the ChiP-IT High Sensitivity Kit (Active Motif) according to the manufacturer’s instructions. Chromatin was immunoprecipitated with 2 μg of specific antibodies. Reverse crosslinking of DNA was followed by DNA purification with the ChIP DNA Clean & Concentrator kit (Zymo Research). The amount of DNA immunoprecipitated with each antibody was measured in duplicate by qPCR. Primers sequences are listed in Supplemental Experimental Procedures.

### Endogenous Immunoprecipitations and Western Blot Analysis

MCF-7 cells were harvested by rinsing with PBS, and total extracts were incubated at 4°C overnight with the antibodies in IPH buffer (50 mM Tris [pH 8], 150 mM NaCl, 5 mM EDTA, and 0.5% NP-40 v/v). Standard procedures were used for western blotting. For control western blotting of RNAi-treated cells, antibodies against EMSY, ETS-1, KDM5B, and β-tubulin were used. Finally, membranes were incubated with secondary antibodies conjugated with horseradish peroxidase, and signals were visualized with enhanced chemiluminescence (GE Healthcare). Images were scanned and processed with Adobe Photoshop CS, and slight contrast was applied equally across the entire images.

### Invasion and Migration Assays

2.5 × 10^4^ cells were plated in serum-free media in the upper chamber with either non- or Matrigel-coated membranes (24-well insert; pore size, 8 mm; BD Biosciences) for transwell migration and invasion assay, respectively. The bottom chamber contained DMEM with 10% FBS. After 24–48 hr, the bottom of the chamber insert was fixed and stained with crystal violet. Cells on the stained membrane were counted under a microscope. Each membrane was divided into four quadrants, and an average from all four quadrants was calculated. Each assay was performed in biological triplicates.

### Statistical Analysis

Statistical analysis was carried out with Microsoft Excel or in the R statistical computing language in order to assess differences between experimental groups. Statistical significances (Student’s t test) are expressed as a p value.

## Author Contributions

E.V. conceived and coordinated the study, designed and carried out experiments, interpreted data, assembled figures, and wrote the manuscript. T.K. designed experiments, interpreted data, and wrote the manuscript. C. Curtis, A.G., and C. Caldas performed experiments, analyzed data, and edited the manuscript. V.D. and M.E. contributed to animal work. A. Villanueva and A. Vidal performed the pathological analysis. S.R. contributed to TF binding site identification. S.A. provided access to tumor data.

## Supplemental Information

Document S1. Supplemental Experimental Procedures, Figures S1–S5, and Tables S1–S3
