# The Breast Cancer Oncogene EMSY Represses Transcription of Antimetastatic microRNA miR-31

**DOI:** 10.1016/j.molcel.2014.03.041

**Published:** 2014-04-10

**Authors:** Emmanuelle Viré, Christina Curtis, Veronica Davalos, Anna Git, Samuel Robson, Alberto Villanueva, August Vidal, Isaia Barbieri, Samuel Aparicio, Manel Esteller, Carlos Caldas, Tony Kouzarides

(Molecular Cell *53*, 806–818; March 6, 2014)

Due to an error during the submission of this manuscript, we inadvertently omitted the name of one of the authors, Isaia Barbieri, from the author list. The name now appears in the online version of the manuscript.

