# Supplementary material for: The Breast Cancer Oncogene EMSY Represses Transcription of Antimetastatic microRNA miR-31
Source: Mol Cell. 2014 Mar 6;53(5):806–18. doi: 10.1016/j.molcel.2014.01.029 (PMC3988886; doi:10.1016/j.molcel.2014.01.029)
Supplement: Document S1. Supplemental Experimental Procedures, Figures S1–S5, and Tables S1–S3 [file mmc1.pdf]

Molecular Cell, Volume *53*

Supplemental Information

The Breast Cancer Oncogene EMSY
Represses Transcription
of Antimetastatic microRNA miR-31

Emmanuelle Viré, Christina Curtis, Veronica Davalos, Anna Git, Samuel Robson, Alberto Villanueva, August Vidal, Samuel Aparicio, Manel Esteller, Carlos Caldas, and Tony Kouzarides

Figure S1, related to Figure 1, Viré *et al*.

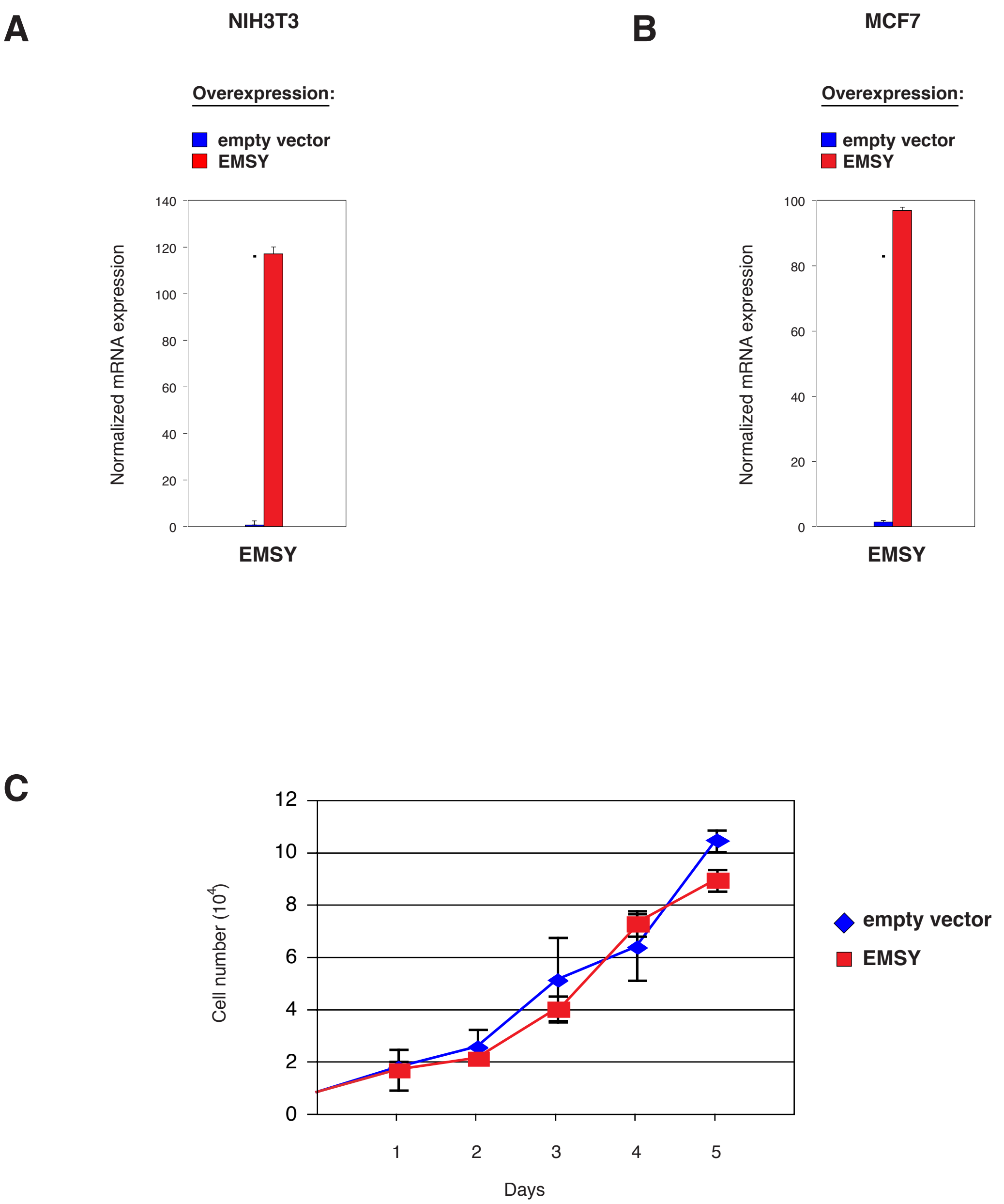

Figure S1

(A) Mouse fibroblast NIH3T3 cells stably overexpressing human EMSY. Cells were transfected with the indicated vectors. Cellular mRNA was isolated and the level of endogenous EMSY expression was measured by RT-qPCR. Gene expression change, normalized with respect to B2M, is presented as relative fold-change compared to the values of empty vector transfected cells (set at 1.0). Each value is the average of three independent experiments with error bars displaying S.D. of the mean.

(B) Characterization of the EMSY overexpression in human MCF-7 cells presented as in (A).

(C) Growth curve for MCF-7 cells overexpressing EMSY or empty vector. MCF-7 cells stably overexpressing EMSY or an empty vector were counted every 24 hours over for 5 days. The means of cell numbers are expressed as means +/- S.D. from triplicate wells. Experiment was repeated three times and the data from a representative experiment is shown.

Figure S2, related to Figure 2, Viré *et al*.

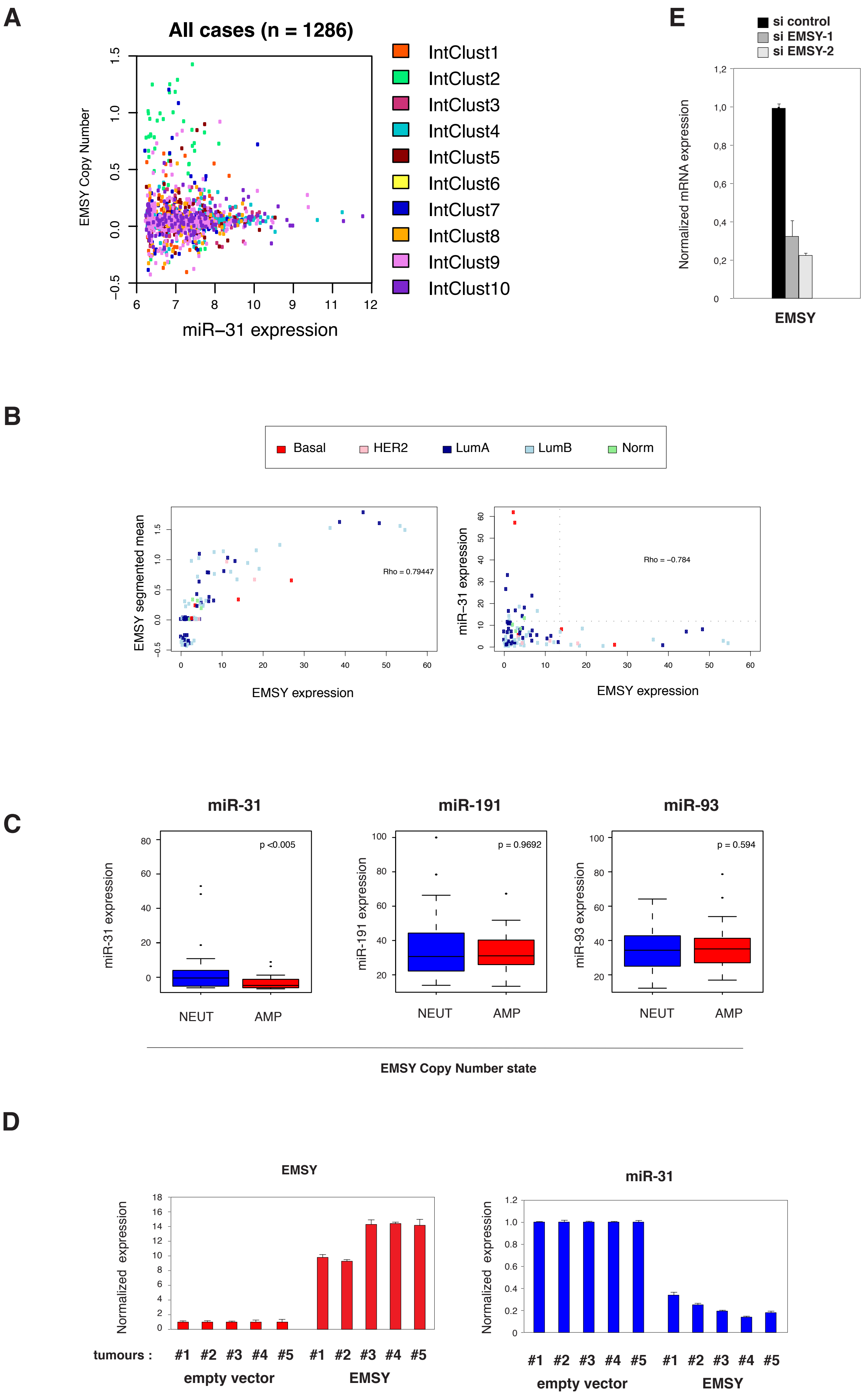

Figure S2

(A) Comparison of EMSY copy number (CN) versus miR-31 expression using scatterplots where samples are colored according to the integrative cluster subgroups. This plot illustrates that only the tumors with moderate miR-31 expression exhibit EMSY Copy Number gain/amp.

(B) Correlation between EMSY and miR-31 expression in 98 tumours profiled by qPCR.
Left: scatterplot illustrating the correlation between EMSY copy number (segmented mean) and EMSY expression for 98 tumours from the METABRIC cohort. Each point represents a tumour, which has been colored according to intrinsic subtype based on the PAM50 classifier. Right: scatterplot illustrating the anti-correlation between miR-31 and EMSY expression levels. Here, the correlation was evaluated using only the 85% (top 15%) of either EMSY or miR-31 expression levels as demarcated by dotted lines.

(C) A representative subset of 98 primary tumours was selected, and miR-31 expression levels were profiled by qPCR. Boxplots illustrating the distribution of miR-31 expression for EMSY amplified (AMP ; n=28 ; red) versus EMSY copy number neutral (NEUT ; n=30, blue) cases. miR-191 and miR-93 are used as controls. Wilcoxon rank sum test was used to evaluate whether expression levels varied significantly depending on EMSY copy number state.

(D) EMSY and miR-31 levels in mammary fat pad tumours. Tumours from the mammary fat pad experiment (Figure 1C) were collected and RNA was extracted using the miRVANA kit (Ambion), following manufacturer's instructions. The level of human EMSY and miR-31 expression was measured by RT-qPCR. Results were normalized to the B2M and RNAU5A for EMSY and miR-31 respectively. Each value is the average of two independent experiments.

(E) Characterization of the EMSY knock-down in MCF-7 cells. MCF-7 cells were transfected with the indicated siRNAs. Cellular mRNA was isolated and the level of endogenous EMSY expression was measured by RT-qPCR. Gene expression changes, normalized with respect to B2M, are presented as relative fold-change compared to the values of cells transfected with empty vector (set at 1.0). Each value is the average of three independent experiments with error bars displaying S.D. of the mean.

Figure S3, related to Figure 3, Viré *et al*.

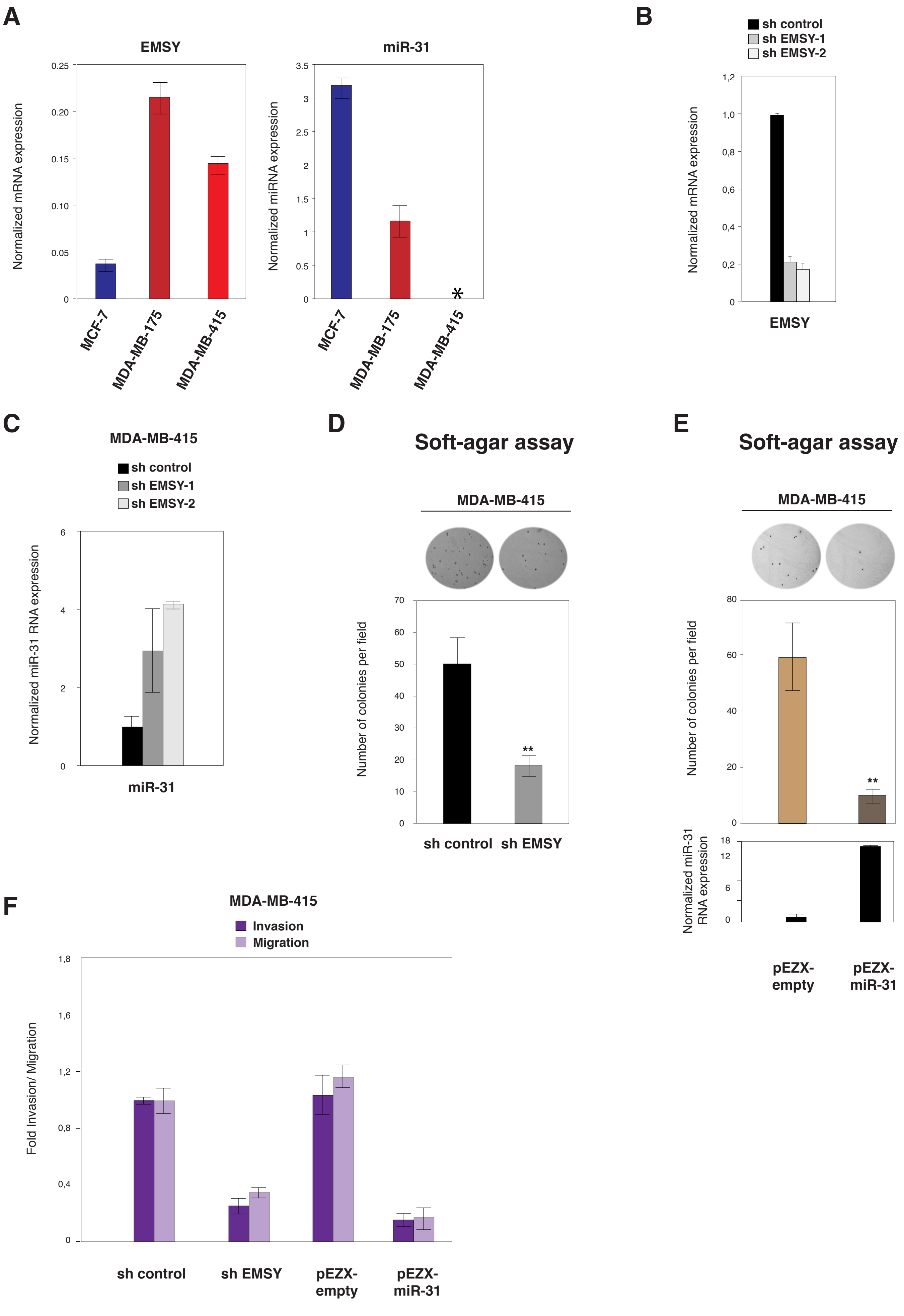

Figure S3

(A) EMSY and miR-31 levels in three cell lines used in the study. Cellular mRNA was isolated from MCF-7, as well as from two lines harbouring amplification of the EMSY gene: MDA-MB-415 and MDA-MB-175 cells. The level of EMSY (left panel) and miR-31 (right panel) expression was measured by RT-qPCR. Results were normalized to the B2M and RNAU5A for EMSY and miR-31 respectively. Each value is the average of two independent qPCR with error bars displaying S.D. of the mean. * indicates undetected levels by qPCR.

(B) Characterization of the EMSY knock-down in MDA-MB-415 cells. Cells were stably transfected with the indicated vectors. Cellular mRNA was isolated and the level of endogenous EMSY expression was measured by RT-qPCR. Gene expression change, normalized with respect to B2M, is presented as relative fold-change compared to the values of empty vector transfected cells (set at 1.0). Each value is the average of three independent experiments with error bars displaying S.D. of the mean.

(C) EMSY knock-down in MDA-MB-415 cells leads to up-regulation of miR-31. Cellular mRNA was isolated MDA-MB-415 cells stably depleted for EMSY. The level miR-31 expression was measured by RT-qPCR. Results were normalized to the RNAU5A. Each value is the average of two independent qPCR with error bars displaying S.D. of the mean.

(D) MDA-MB-415 cells were stably depleted for EMSY and cells were grown in soft-agar for 21 days. Colonies were stained with thiazolyl blue tetrazolium bromide and counted. Quantification of the colony number from three independent soft agar assays is shown. Representative pictures of the colonies are shown.

(E) Exogenous expression of miR-31 in MDA-MB-415 cells decreases their ability to grow in soft-agar and form colonies. Assay was performed as in (D). Each value is the average of three independent experiments with error bars displaying S.D. of the mean. The level of miR-31 (bottom panel) expression was measured by RT-qPCR.

(F) miR-31 expression phenocopies EMSY depletion in MDA-MB-415 cells. Boyden chamber assay was used to monitor the invasion and migration capability of the cells. Each value is the average of three independent experiments with error bars displaying S.D. of the mean.

Figure S4, related to Figure 4, Viré et al.

A

	miRNA genomic location			identified TSS from 5'RACE
Name	Chr	Start End	Strand	
hsa-mir-31	chr9	21512114-21512184	-	21554309 = MIR31HG
hsa-mir-181a-2	chr9	127454721-127454830	+	127420746 = MIR181A2HG
hsa-mir-198	chr3	120114515-120114576	-	120170100 defined by FSTL1 TSS

B

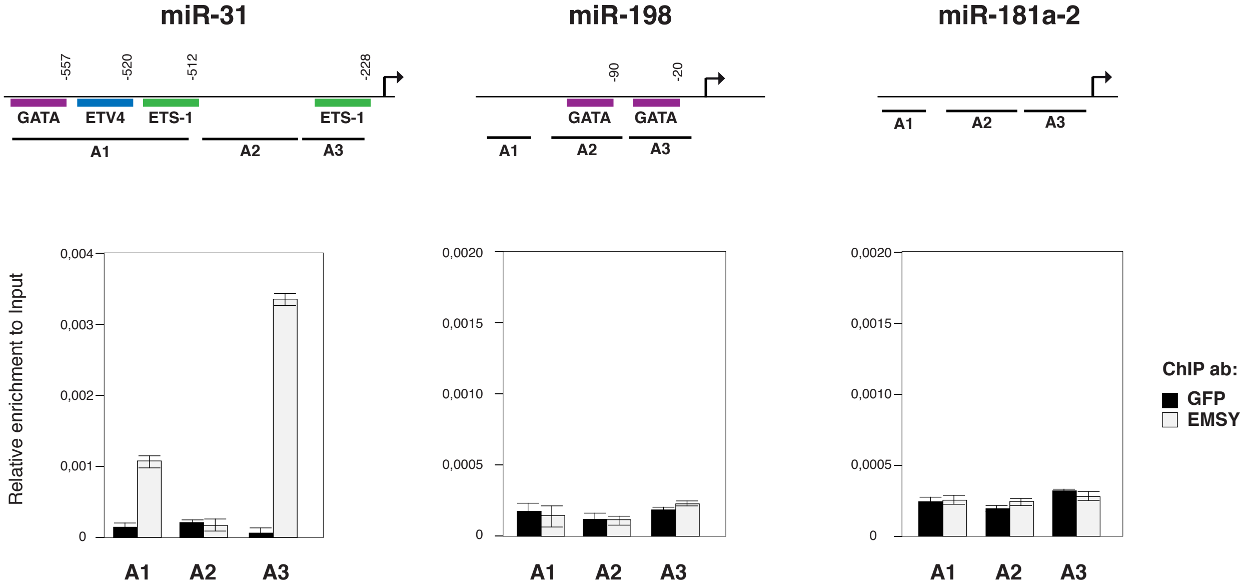

C

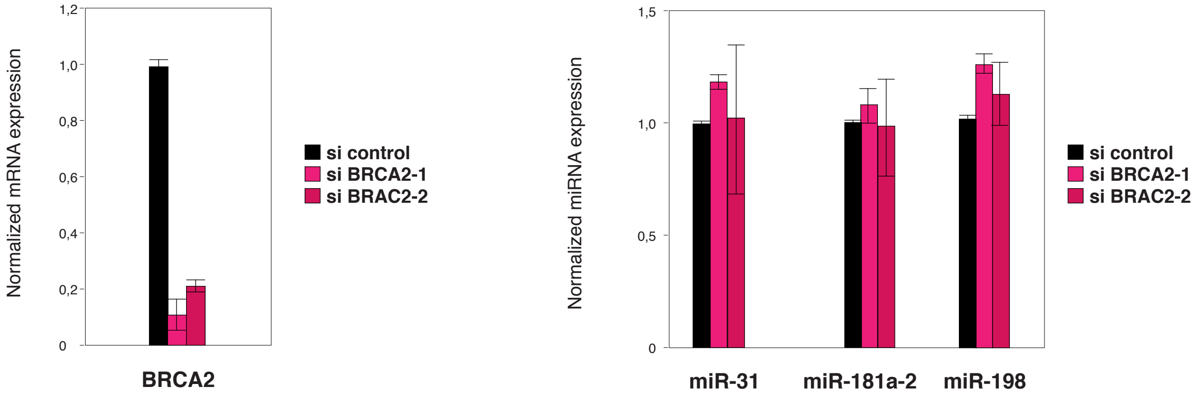

Figure S4

(A) Determination of the transcription start site for the primary transcripts of the miRNAs using 5' RACE. Total RNA extract from MCF-7 cells and was treated for GeneRacer PCR according to the manufacturer's protocol. After cloning and sequencing, the transcription start site was identified. These results are consistent with the mapped TSS available in the miRSTART database.
(B) EMSY specifically associates with the miR-31 promoter. Chromatin from MCF-7 cells treated with control siRNA or EMSY siRNA was immunoprecipitated with an anti-EMSY (white bars) or anti-GFP (black bars) antibody. The data have been normalized to "Input" amplification levels for each amplicon. Each amplicon was analysed in duplicate. Experiments were repeated at least two times with similar results. A representative example of a ChIP analysis is shown.
(C) BRCA2 does not affect miR-31 expression. MCF-7 cells were transfected with two different siRNAs as indicated. Cellular mRNA was isolated and the level of endogenous BRCA2 expression was measured by RT-qPCR (left panel). The expression level of BRCA2 in cells treated with control siRNA was assigned a value of 1.0 following normalization to the B2M house-keeping gene whose expression did not change following treatments with siBRCA2. Each value is the average of three independent experiments with error bars displaying S.D. of the mean. miRNA expressions were assayed by RT-qPCR (right panel). Gene expression changes, normalized with respect to RNU5A small RNA expression, are presented as relative fold-change compared to the values of control-siRNA-treated cells (set at 1.0). Each value is the average of two independent experiments with error bars displaying S.D. of the mean.

Figure S5, related to Figure 5, Viré et al.

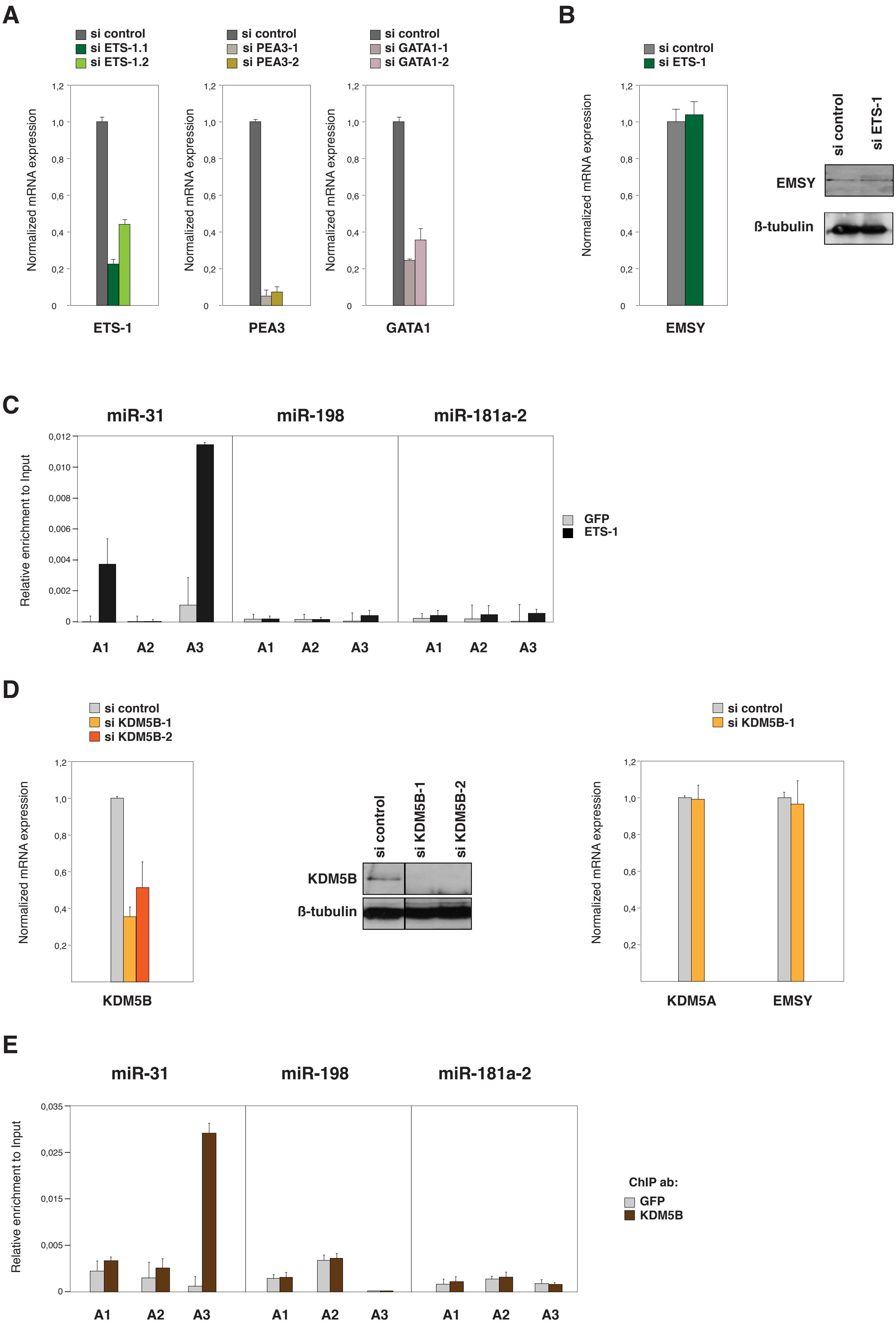

Figure S5

(A) MCF-7 cells were transfected with each of the indicated siRNAs. Cellular mRNA was isolated and the levels of endogenous ETS-1, ETV4 (PEA3), and GATA1 expression were measured by RT-qPCR. Gene expression changes, normalized with respect to B2M, are presented as relative fold-change compared to the values of control-siRNA-treated cells (set at 1.0). Each value is the average of three independent experiments with error bars displaying S.D. of the mean.

(B) ETS-1 depletion does not affect EMSY expression. Endogenous EMSY is not affected by ETS-1 knockdown. EMSY expression was measured by RT-qPCR in MCF-7 cells depleted for ETS-1 (left panel). Gene expression changes, normalized with respect to B2M, are presented as relative fold-change compared to the values of control-siRNA-treated cells (set at 1.0). Right panel: representative Western blot analysis of EMSY expression in MCF-7 cells depleted for ETS-1. ß-tubulin was used as a loading control.

(C) Validation of the ChIP analyses with ETS-1 antibody. ChIP analysis of ETS-1 associated with the miR-31, miR-198, and miR-181a-2 promoters. MCF-7 cells were harvested, and used for ChIP experiments using anti-ETS-1 (white bars) or anti-GFP (black bars) antibodies. The precipitated DNA fragments were subjected to qPCR analysis with specific primers corresponding to 3 amplicons encompassing the regulatory region of each miRNA as indicated. ChIPs performed with anti-GFP serve as negative controls. The data have been normalized to "Input" amplification levels for each amplicon. Each amplicon was analysed in duplicate. Experiments were repeated at least two times with similar results. A representative example of a ChIP analysis is shown. Each amplicon was analysed in duplicate each time and error bars represent the S.D. for each amplicon.

(D) Characterization of the KDM5B knock-down. MCF-7 cells were transfected with the siRNAs as indicated. Cellular mRNA was isolated and the level of endogenous KDM5B expression was measured by RT-qPCR (left panel). The expression level of KDM5B in cells treated with control siRNA was assigned a value of 1.0 following normalization to the B2M house-keeping gene whose expression did not change following treatments with si KDM5B. Each value is the average of three independent experiments with error bars displaying S.D. of the mean. Middle panel: representative Western blot analysis of KDM5B expression in MCF-7 cells after transfection with the indicated siRNA duplexes. The imaged was cropped between lane 1 (si control) and lane 2 (siKDM5B-1) as indicated by the black vertical line. The blot was then stripped and re-probed for ß-tubulin which was used as a loading control. Endogenous KDM5A and EMSY are not affected by KDM5B knockdown (right panel). Gene expression was measured by RT-qPCR in MCF-7 cells depleted for KDM5B. Gene expression changes, normalized with respect to B2M, are presented as relative fold-change compared to the values of control-siRNA-treated cells (set at 1.0). Each value is the average of three independent experiments with error bars displaying S.D. of the mean.

(E) Validation of the ChIP analyses with KDM5B antibody. ChIP analysis of KDM5B associated with the miR-31, miR-198, and miR-181a-2 promoters. MCF-7 cells were harvested, and used for ChIP experiments using anti-KDM5B (brown bars) or anti-GFP (white bars) antibodies. The precipitated DNA fragments were subjected to qPCR analysis with specific primers corresponding to 3 amplicons encompassing the regulatory region of each miRNA as indicated. ChIPs performed with anti-GFP serve as negative controls. The data have been normalized to "Input" amplification levels for each amplicon. Each amplicon was analysed in duplicate. Experiments were repeated at least two times with similar results. A representative example of a ChIP analysis is shown. Each amplicon was analysed in duplicate each time and error bars represent the standard deviation for each amplicon.

.

Table S1 , Viré *et al*.

	GeneName	fold change
downregulated	hsa-miR-22-3p	-20.9
	hsa-miR-140-5p	-10.98
	hsa-miR-210	-5.77
	hsa-miR-328	-4.19
	hsa-miR-489	-3.33
	hsa-miR-132-3p	-2.91
	hsa-miR-222-3p	-2.88
	hsa-let-7c	-2.57
	hsa-miR-10b-5p	-2.43
upregulated	hsa-miR-93-5p	2.22
	hsa-miR-497-5p	2.57
	hsa-miR-31-5p	2.84
	hsa-miR-340-5p	3.15
	hsa-miR-96-5p	4.02
	hsa-miR-7-5p	5.89
	hsa-miR-98-5p	8.13
	hsa-miR-152	12.93
	hsa-miR-200a-3p	17.22
	hsa-miR-200b-3p	25.38
	hsa-miR-200c-3p	26.33
	hsa-miR-195-5p	41.5
	hsa-miR-16-5p	43.86
	hsa-miR-193b-3p	46.8
	hsa-miR-186-5p	57.8
	hsa-miR-17-5p	58.96
	hsa-miR-182-5p	68.14
	hsa-miR-203a	70.65
	hsa-miR-15b-5p	73.27
	hsa-miR-15a-5p	74.8
	hsa-miR-19a-3p	83.16
	hsa-miR-19b-3p	87.67
	hsa-miR-181b-5p	95.54
	hsa-miR-18a-5p	113.09
	hsa-miR-181d	125.97
	hsa-miR-181c-5p	158.03
	hsa-miR-181a-5p	181.94

Supplementary Table 1

38 miRNAs are found significantly deregulated in MCF-7 cells depleted from EMSY (9 down-regulated in red and 29 up-regulated in green). The expression of 88 miRNAs known or predicted to alter their expression during breast cancer initiation/progression (Human Breast Cancer miScript miRNA PCR Array, Qiagen) was analysed by qPCR. Gene expression changes are ranked based on relative fold-change compared to the values of cells transfected with empty vector (set at 1.0). p-value is < 0.05.

Table S2 , Viré *et al*.

METAB RIC Cohort: CN, Expression (*n* = 1980)					
	**AMP**	**GAIN**	**NEUT**	**HETD**	**HOMD**
**ER status**					
pos	72	111	1217	98	0
neg	6	29	402	2	0
null	1	2	39	1	0
**PAM50**					
Basal	3	28	297	1	0
HER2	9	14	210	7	0
LumA	23	38	619	38	0
LumB	43	52	342	51	0
Normal	1	9	186	3	0
NC	0	1	4	1	0
**IntClusters**					
IntClust 1	3	17	103	16	0
IntClust 2	44	10	13	5	0
IntClust 3	2	8	272	8	0
IntClust 4	0	11	322	10	0
IntClust 5	4	12	171	3	0
IntClust 6	9	12	54	10	0
IntClust 7	3	12	153	22	0
IntClust 8	4	21	256	18	0
Intclust 9	8	18	112	8	0
Intclust 10	2	21	202	1	0
**Total**	**79**	**142**	**1658**	**101**	**0**

METAB RIC Cohort: mi RNA, CN, Expression (*n* = 1283)					
	**AMP**	**GAIN**	**NEUT**	**HETD**	**HOMD**
**ER status**					
pos	45	75	809	57	0
neg	5	15	245	1	0
null	0	1	30	0	0
**Total**	**50**	**91**	**1084**	**58**	**0**

Supplementary Table 2: the METABRIC cohort of samples

Table S3 , Viré *et a*l.

	GeneName	decideTests	coef	t.test	lods	p.value	adj.P.Val	logFC	AveExpr	ProbeUID
downregulated	hsa-miR-99a	-1	-0.78198069	-3.91260805	0.63422421	9.67E-05	0.0184794	-0.78198069	11.1795819	4404
	hsa-miR-125b-	-1	-0.19092694	-3.90106847	0.59015081	0.0001014	0.0184794	-0.19092694	6.69882456	2895
	hsa-miR-31	-1	-0.4424558	-3.89572621	0.56978987	0.00010363	0.0184794	-0.4424558	7.31123424	1541
	hsa-miR-378*	-1	-0.18161713	-3.77220613	0.10659761	0.00017018	0.02855939	-0.18161713	6.6961028	346
	hsa-miR-146b-	-1	-0.35649555	-3.71163204	-0.1152345	0.00021593	0.03422518	-0.35649555	7.52241174	6713
	hsa-miR-505*	-1	-0.14972527	-3.61583134	-0.45891619	0.00031255	0.04246187	-0.14972527	6.62399204	2669
upregulated	hsa-miR-326	1	0.44557275	10.1402399	41.8431735	3.45E-23	6.18E-20	0.44557275	6.71557113	6816
	hsa-miR-338-3	1	0.85457012	7.036815	17.1079168	3.40E-12	2.42E-09	0.85457012	7.39595114	7170
	hsa-miR-887	1	0.33071999	4.33940924	2.35303497	1.56E-05	0.00739939	0.33071999	6.85344988	5575
	hsa-miR-4254	1	0.07020222	4.23649055	1.92279289	2.45E-05	0.0077813	0.07020222	6.29697977	7708
	hsa-miR-29c*	1	0.50129975	4.19003049	1.73184723	3.01E-05	0.00824951	0.50129975	8.97717395	137
	hsa-miR-887	1	0.09989008	3.69419596	-0.17843882	0.00023111	0.03470282	0.09989008	6.35798871	8182

Supplementary Table 3

Using the METABRIC dataset, EMSY amp versus neutral cases were compared.
12 miRNAs are found significantly deregulated (6 down-regulated – in red- and 6 up-regulated – in green- ; Benjamini and Hochberg adjusted P-value < 0.05).
The miRNAs have been sorted by their adjusted p-value.

SUPPLEMENTAL MATERIALS

Primers for 5'Race:

RACE	First PCR	Nested PCR
miR-31	GGAAATCCACATCCAAGGAA	ATGTTGGCATAGCAGGTTCC
miR-198	TGTCAGATGGCCAAAAACAA	CCAGAGGGGAGATAGGTTCC
miR-181a-2	CAGTCAACGGTCAGTGGTTTT	CGACAGCGTTGAATGTTCCT

Transient and stable transfections. Commercially available validated short hairpin RNA molecules were used to knock-down either EMSY, BRCA2, ETS-1, PEA3, GATA1, KDM5B or control siRNA. miR-31 inhibitor was purchased from Qiagen. Transfection was performed using either Lipofectamine RNAiMax (Invitrogen) or Ribocellin (BioCellChallenge) according to the manufacturers's instructions. Plasmids for stable expression (depletion and overexpression) were obtained from GeneCopoeia. Plasmids for miR-31 sponge and control were obtained from Addgene (Plasmid 22694: pBABE-puro-miR-31 sponge) and plasmids for miR-31 overexpression (pEZX) were purchased from GeneCopoeia. For generation of stable cell lines, transfection was performed using the FuGene6 transfection reagent (Roche Diagnostics) and followed the protocol provided by the manufacturer. At 24 h post transfection, cells were splitted and stable clones were selected by the addition of Puromycin (1 µg/ml) to growth medium.

siRNAs references

EMSY (Hs_C11orf30_5, Hs_C11orf30_6, Hs_C11orf30_7, Hs_C11orf30_8), BRCA2 (Hs_BRCA2_6, Hs_BRCA2_7), ETS-1 (Hs_ETS1_3, Hs_ETS1_5), PEA3 (Hs_ETV4_2, Hs_ETV4_8), GATA1 (Hs_GATA1_1, Hs_GATA1_3), KDM5B (Hs_KDM5B_3, Hs_KDM5B_6) or control siRNA (Ctrl_GFP_3) (Qiagen).

Antibodies

EMSY (A-300-253A, Bethyl), ETS-1 (C-20, Santa Cruz), H3K4me3 (17-614, Upstate), KDM5B (ab50958, Abcam), RNA polymerase II (05-623, Millipore), anti-SUZ12 (ab12073, Abcam), GFP (ab290, Abcam), ß-tubulin (ab6046, Abcam), and rabbit IgG (Upstate)

Promoter sequences for all three miRNAs annotated for the relevant motifs

TSS: **bold underline**
ETS-1 motif: green
ETV4(PEA3) motif: blue
GATA1 motif: purple

hsa-mir-31

CCCCACCCTTCAACTCGTAGAATATTTTTTGGTGGTAACTAGACTGACCTTTGCC
AAAATGTAATTACATTCAGAGAGAAATACAATTTTCCCCTTCAAATCCAGGTGAAA
GGCTACCATGCTGGGCTTGCGGAGGGCATAGTTGAGTAGGACAGCCAGAACTT
CTGGCTAGGTCATCGGGTCGGATTACCCTGCCATGTACCCCTCAGTATTTACTTC
CAGAGATGGGCAACTTACTATAGTCTTCATGTTTAAGGAATTCAAACCCAAATGA
AAAGTGTTGTGAAAGCATAAATATCTCTGCTTGTATACTACAGTACTGAATATGAA
ACCACATATTGCAATAAAAATAACTAACGATGGCCCATGCATTTCACAAGGCGCT
CTCATACACCTGAAGGGGCAGTGGAAAGGTTCAGAGCACCCAGGCTGTCCCAA
AGAGGGCGGAATCAAATCTGGGCGCAGGTGGGTACCCGGAAAAAATTCTTGGT
GGAGATGGCATCTGAGAGAGGCGTCGAAGGACACGGAGGGTTGAGGATAGGA
GAGAGGCCACTCCAGATGGAAAAACACCCTCGTTTTCCAAATGCCAGTTATACC
ACGGGGCCTGTGATAGCCGCCACGTTTCTAGAAGACCCCTAGAAGAGCAGGGG
TCCGACCAAGGAAGTA**CTTCCG**CTGTTCAATTTGCTAAGTTTGCATTCGGAGAGT
ACAAGGGCGAGCTCTGGCTCTTGCAGGTGGACTCCCTCTCCCTTAGCTCTGACT
GCGAGAAGCTCCCACCCGCAAAGGGCTACTGTGCCAAAAGCGAGCGTAGTGGC
CGCCGCTTCCCCCACAAAATTCCGTCACTCGGGAGCTGCGTTGCAGTGGAGAAA
CCTGGGTCCCGTCCGGGCCCAAGGGAGGAGGCTCAGCACGGCCGCTCCCGCA
GCCCCGTCCAGGCGCCTCTTCCCTCCCCCTCC**TTTCCG**CGGCGGTCGCGGCGG
CGGCTGGAGCGGGAGCCGGGGGCGGCGAGGGGCGGAGCCGGGGCGGAGCCA
CGGCGCTGGCGGCCCCGGCGGCGGCGGCAGGTGTGGATGGGGCGAAGGTGC
GGGAACGTCCACTCCCGCGCGCCCCTCTCGGGGACCAGGGCGGCCTCCCAGG
AGGAGCTTGGTGAGCAGCTGCGACCTGTGCATAACTTGGGGCGCCGCCAGG**GG**
CTCGCAGGTTCCACGTCCGGCGCCTGGAGAAGGAAGACGCGCGTCCCCGGCC
GCGGCGCCGA

Amplicon1:underline
Amplicon2:double underline
Amplicon3: wave underline

hsa-mir-181a-2

AAACAAAGCAACTGCCATGTACTAAACAAAAATGTCACAAATGACAGTCCTGTAT
AATGACCTGGTTTCCCAGTGGCTCAGAAAGATGGAAAATAAACCCCAAGGACCC
TAGTTCAGACAGACTGAAGAGAGCAGACCGTGGCTGTCTTGCATGATTAAAGGC
TATGTCACAGCTCAGTAGGGTCCATGCTTTCATAGCTTCCCCCCCCAGCAACC
CCTAATGTGACTAGGACTGGGACAAGAGTGACAGTCTCACAAAGGGGATAAAAA
GACATAAAGCTTCTTCCTCATACATCCAAACACTCCAGAATCTAAAACTTCACTTC
TCTGTTGTCCATCTATCCAATTTTCATTAAGAGCAATTTATATCATATAGAGGCTT
CAAAGTCAAATATGCCACTTTTATTTTTACCTTATCAAACCTATAAAATCAAGACC
AAGGGTTGGTCTGGATATGAAGCTTGGTTAGGATCACAATAAATTTTCAAATCAA
ATAATTGGTTTCAGTTAGAGCCAAGTTTATCAACAATAGGGTGATTTAGGATTCA
GGAAAAATCGGCTGCCTATTTGTATTTCAGAAAGACTAGTATTTCTTCTGACACA
AGTAGAGAGGTGTAAGCAACCTCTGGGTGGGTAATGCCACCAAACAAAAGCAAC
ACCTTCAAAGTCTCTCAGTGACTAGAGGCAGCCAGACACTCAAACTGCCAGTCC
TGAATTACGGCCTCTGAATGGTGAGGGCTTCACATTTCACCGATCAGGGCACAG
CCCAATCAGAAGACAAACCCCTGCACTGCCAGGGACCAGCAAATCCGCTTTCTT
CCAGTGTGCACATGACTAAATGTGCTTCTGTCCATCATCTGGGTCATATATAGTT
CAATGCGAGCTGAGCAGACAGGGCTGCAAGGAAATCTGGCGCGGTTCAATA**CC**

TCGTCTAGCCTGGGTTCCAGTATCTAATTTTTTTTTTGTTTTAACTGACAAACTCA
TTTCTCTA

Amplicon1:underline
Amplicon2:double underline
Amplicon3: wave underline

hsa-mir-198
GCCACTGTCTTCAAGATTAAGAGGGCGAAGCTGCCTGAAAGTAGAACTATTCTC
AAATCATTTGCTAGTCAGGCATTCCCCTGTTCAGACGTGTGCAGCAGAACTGAC
AAGGCAGGGAATGGTCTCAGTGTATGCCACCTGAGAATGTGGGGGTATTGACAG
GCACTGATCTGTTGGTGGCTCATCCCTTCCCTCCCATTCACTGGTGGCAGAAAG
CCAGCCCCACCTCATTCACCACTCAACCCAGGGGTAGTGGTGGTTGCATGAAGG
AGAATGTGATTTCCCCAGCTTCTCATCCTGACTGGCTGGGCAGGCAAAATCACG
AAAGACCTCCAGACTCAAGGCTACTGGGCTAGATGCTGTGACAAAACCAACAAA
GACTGCAGGAGGCACGATGGGGTCATGGGGCTGGCTGGGGTAAGGCAGGGAT
GGAACCAGACAGTCCACCCTCCAGGCCATGTCTACCACATTATGCAGAGAACTC
ACACCCACAAGTCTTGTAACAGTACAATTCTTATAGTTGGTGAATGTTTTATACTC
AAGATTAGGGGAAAAAAGCAAAAAGAAAGAAGAGGCTTTCGAGCTGAGGTGCTG
TATCCAATACAGCTAGTCTGCTGCCACCCAGAGGCTGCTCTGCAAACTCCAGGT
CATTATTTTCTGTCCCAAGCCAGCTTGCTGGTCATGCAGGGCTCCCATTGGTCTA
GAAGCCGTTCTCAGCTCACATTTCATTGGTTCACATGCTGTTGATAAACCAATTC
TTTTTTGAATGTTGCTAAGGCGAATTCAAGAAAGCTAGCAGGCGGGGAAGAAAG
AGGAAGTAATTCCCAGAAGTCCGCGGGGATTGGAGAGCTTTTGTTTTCCTAAAC
AGAGAGTGGAGATAAAAATCAAGGATATTCTCCAGGCAGGGCCCTCTTGTGGAA
ACTGTGCCTGACAATGTAAGGCAACACGAAAAAGGAAGAGGAAAAGGTTTTTGC
TGACGAGTTGTTCCCATGAGTTATGGACACACAAATGCATAAAAAAGCATGCTCA
GACACATGCCTGCACTCAAAGACACCCGCACACAGCTCCACAGGCAAACACCCA
CAAACCTCCACTCACTAGTCTCTGTCCTTAACTAGTAATTTTCATTCTTTAAGGCT
CACATGTCCTCATCTATAAAGTAGGGGTAAATGCCAACCCTGCCCTGCGGAGAT
TCAGTGAGATACATGTGGAAGTACCAACTGTAAAACAAGCTGCATATGAAACCTG
TTATTCATGGGAATGCCTTATAGAGCTACTTGCAGAAAAGCCTGTGGCATGTATG
GAACCTCCTTTTAACTTACATTGTTGTTTTTAGTAAGTATTAAAAACGGATTGAAT
TAAACCAAGTCATTTTTCAGGAATAGTTCTCAAAAATCCATTTGAAGAAACAGATA
ACTTTTGAGTTATAACAACTAATATTTGAATAGTACAATTTACAAAATGTTTTCATA
CACATGATTTTGTCTTATAGGGAAACCCTGCAAGAATAGGTATATTATTATTAACC
CTATTCCATAGATGAGGAAATTGAAGACCAGAAAGGTCAGATAACTTGCCCAAGT
TTCCAAAGCTAGAAATGAGAGGAGCTAGGATTCCAACTCGTATCTTCAACCACTG
ATAACTAAACTCCCATTTATAAAT**ATCCG**GCATCCT

Amplicon1:underline
Amplicon2:double underline
Amplicon3: wave underline
